# New data on two remarkable Antarctic species *Amblydorylaimus isokaryon* (Loof, 1975) Andrássy, 1998 and *Pararhyssocolpus paradoxus* (Loof, 1975), gen. n., comb. n. (Nematoda, Dorylaimida)

**DOI:** 10.3897/zookeys.511.9793

**Published:** 2015-07-02

**Authors:** Milka Elshishka, Stela Lazarova, Georgi Radoslavov, Petar Hristov, Vlada K. Peneva

**Affiliations:** 1 Institute of Biodiversity and Ecosystem Research (IBER), Bulgarian Academy of Sciences, 2, Gagarin Street, 1113 Sofia, Bulgaria

**Keywords:** 18S rDNA, D2-D3 28S rDNA, morphology, new geographic records, nomenclature, SEM, taxonomy

## Abstract

The taxonomic position of two antarctic dorylaimid species *Amblydorylaimus isokaryon* (Loof, 1975) Andrássy, 1998 and *Pararhyssocolpus paradoxus* (Loof, 1975), **gen. n.**, **comb. n.** are discussed on the basis of morphological, including SEM study, morphometric, postembryonic and sequence data of 18S rDNA and the D2-D3 expansion fragments of large subunit rDNA. The evolutionary trees inferred from 18S sequences show insufficient resolution to determine the assignment of the two species to particular families, moreover *Pararhyssocolpus paradoxus* **gen. n.**, **comb. n.** (=*Rhyssocolpus paradoxus*) previously regarded as a member of Nordiidae or Qudsianematidae, showed distant relationship both to *Rhyssocolpus vinciguerrae* and *Eudorylaimus* spp. The phylogram inferred from 28S sequences revealed that *Amblydorylaimus isokaryon* is a member of a well-supported group comprised of several *Aporcelaimellus* spp., while, no close relationships could be revealed for the *Pararhyssocolpus paradoxus* **gen. n.**, **comb. n.** to any nematode genus. On the basis of molecular data and morphological characteristics, some taxonomic changes are proposed. *Amblydorylaimus isokaryon* is transferred from family Qudsianematidae to family Aporcelaimidae, and a new monotypic genus *Pararhyssocolpus* **gen. n.** is proposed, attributed to Pararhyssocolpidae **fam. n.** The diagnosis of the new family is provided together with emended diagnosis of the genera *Amblydorylaimus* and *Pararhyssocolpus* **gen. n.** Data concerning distribution of these endemic genera in the Antarctic region are also given.

## Introduction

Taxonomic studies on Antarctic nematodes are sparse and current knowledge about species distribution, biogeography and their relationship to the global fauna is still poor (Velasco-Castrillón and Stevens 2014). Almost all Antarctic nematode species have been recorded only from this region (Andrássy 1998a; Maslen and Convey 2006; Convey et al. 2008). This high degree of endemism is probably caused by the long-term isolation and harsh climate of the region (Convey et al. 2008; Nielsen et al. 2011), and indicates that they are glacial survivors rather than post-glacial colonists (Andrássy 1998a; Maslen and Convey 2006; Chown and Convey 2006).

Nineteen species of order Dorylaimida Pearse, 1942 have been recorded from this region which is approximately 0.7% of the known dorylaimid species; all of them being endemic. Regarding the genera distribution a single genus, namely *Amblydorylaimus* Andrássy, 1998 inhabiting Maritime Antarctic is considered endemic.

Molecular studies of free-living Dorylaimida members are increasing (Mullin et al. 2005; Holterman et al. 2006; 2008; Pedram et al. 2009; 2011; Álvarez-Ortega et al. 2013a, b, c; Nedelchev et al. 2014; Peña-Santiago et al. 2015). Nevertheless, Antarctic dorylaimids have received little attention in this respect with only one study of this widespread order in Antarctic. Velasco-Castrillón and Stevens (2014) analysed the morphological and molecular diversity of Antarctic nematodes using the mitochondrial cytochrome c oxidase subunit I (COI) gene.

Here we present data on the morphology, molecular taxonomy and distribution of two dorylaimid species with unclear taxonomic position occurring in the Maritime Antarctic.

## Materials and methods

Samples were collected from Livingston, Nelson and King George Islands by Dr. N. Chipev, Dr R. Mecheva (IBER) and Dr R. Zidarova (Faculty of Biology, Sofia University St. Kliment Ohridski) during regular Bulgarian Antarctic Expeditions (1997–2013). Nematodes were extracted from soil and plant materials by using a Baerman funnel method for 48 or more hours of exposition, killed by gentle heat and fixed in 4% formalin.

For light-microscopy, specimens were processed in anhydrous glycerine by a Seinhorst method (1959) and mounted on permanent slides. Drawings were prepared using an Olympus BX 51 compound microscope with DIC and a drawing tube. Photographs were taken using an Axio Imager.M2 – Carl Zeiss compound microscope with a digital camera (ProgRes C7) and specialised software (CapturePro Software 2.8). Measurements were made using an Olympus BX 41 light microscope with a drawing tube and digitizing tablet (CalComp Drawing Board III, GTCO CalCom Peripherals, Scottsdale, AZ, USA) and Digitrak 1.0f computer program (Philip Smith, John Hutton Institute, Dundee, UK).

Specimens used for SEM observations were rinsed in 0.1 M cacodylate buffer (twice for 10 min), post-fixed in 1% OsO_4_ for 2 h, washed twice for 10 min in 0.1 M cacodylate buffer and dehydrated in an ethanol series (Mutafchiev et al. 2013), immersed in hexamethyldisilazane for 30 min and air dried. They were coated with gold in fine coater JEOL JFS 1200 and examined using a JEOL JSM 5510 microscope at 10 kV.

The location of pharyngeal gland nuclei is presented following Loof and Coomans (1970) and Andrássy (1998b).

### DNA extraction, amplification and sequencing

Genomic DNA was extracted from one male and one female specimen of both species using a standard nematode digestion protocol (Holterman et al. 2006). The specimens used for DNA extraction, amplification and sequencing are from Nelson (*Amblydorylaimus isokaryon* (Loof, 1975) Andrássy, 1998) and King George (*Pararhyssocolpus paradoxus* gen. n., comb. n.) Islands. For further details, see Nedelchev et al. (2014). Identical sequences were obtained from both individuals of the same species. The sequences of both Antarctic species have been deposited in GenBank with the following accession numbers: for the 18S rDNA KM092519 (*Amblydorylaimus isokaryon*) and KM092521 (*Pararhyssocolpus paradoxus* gen. n., comb. n.) and for the D2-D3 rDNA KM092520 (*Amblydorylaimus isokaryon*) and KM092522 (*Pararhyssocolpus paradoxus* gen. n., comb. n.).

### Sequence and phylogenetic analysis

A BLAST (Basic Local Alignment Search Tool) search at NCBI (National Center for Biotechnology Information) was performed using the obtained sequences as queries to confirm their nematode origin and to identify the most closely related nematode sequences. The sequences revealing highest similarity to newly obtained sequences were included in the phylogenetic analyses of both ribosomal gene regions (Griffiths et al. 2006; Holterman et al. 2006; Meldal et al. 2007; Lesaulnier et al. 2008; Pedram et al. 2010; Pedram et al. 2011; Álvarez-Ortega and Peña-Santiago 2012a; 2012b; Donn et al. 2012; Álvarez-Ortega et al. 2013; Nedelchev et al. 2014).

The Multiple Sequence Alignments (MSA) of both gene regions were performed using the Clustal Omega tool (Sievers et al. 2011) via the EBI webserver: http://www.ebi.ac.uk/Tools/msa/clustalw2/. Two datasets (big and small, consisting of 61 and 17 sequences, respectively) were analysed for 18S rDNA. Subsequently, the MSAs were manually optimised and trimmed using MEGA 6 (Tamura et al. 2013). Newly acquired sequence from another Antarctic species *Coomansus gerlachei* (de Man, 1904) Jairajpuri & Khan, 1977 from Nelson Island (accession number KM092523) was used as outgroup species for the big 18S dataset. Otherwise, midpoint rooting was applied for other sequence datasets due to the uncertainties in species identification and non-monophyly of the families Aporcelaimidae Heyns, 1965, Qudsianematidae Jairajpuri, 1965 and Nordiidae Jairajpuri & Siddiqi, 1964, observed in other studies (Holterman et al. 2008). The best-fitting model (General Time Reversible model plus Gamma distribution rates (GTR + G)) of nucleotide substitution for both datasets was estimated using the Bayesian (BIC) and Aikaike Information Criteria (AIC) in MetaPIGA v3.1 (Helaers and Milinkovitch 2010). Subsequently, the phylogenetic reconstructions were performed using the Bayesian Inference (BI) algorithm implemented in MrBayes 3.2.2. (Huelsenbeck and Ronquist 2001; Ronquist et al. 2012). A total of 759 and 1616 positions in the final datasets were used for D2-D3 and 18S rDNA dataset, respectively. The Bayesian MCMC tree searches were run using default heating parameters for 2 000 000 generations with a sample frequency of 1000 generations. The first 25% of the chains discarded as burning and the remaining 75% trees kept to summarise the tree topology, branch lengths, and posterior probabilities (PP) of branch support. Convergence diagnostic values were calculated every 1000 generations with a predefined stop value equal to 0.01. A single strict consensus tree was visualised using FigTree v1.4.0 graphical viewer (http://tree.bio.ed.ac.uk/software/figtree/). Posterior probabilities values of ³0.80 were considered as credible support values for nodes.

## Taxonomy

### Amblydorylaimus isokaryon

Taxon classificationAnimaliaDorylaimidaQudsianematidae

(Loof, 1975) Andrássy, 1998

Figures 1 , 2 , 3 , 4 , 5 , 6 , 7 , 8 , 9 , 10 , 11 , 12 , 13 , 24 , 25 , 26

Eudorylaimus isokaryon Loof, 1975

#### Material examined.

Twenty-two females, nineteen males and thirteen juveniles (J1-J4) collected from three islands from Maritime Antarctic (Table 1).

**Table 1.** Origin of the examined materials of *Pararhyssocolpus paradoxus* gen. n., comb. n. and *Amblydorylaimus isokaryon*.

Site description	Collection year	Abbreviation	Nematode species
**King George Island (KGI)**			
*Fildes Peninsula* Soil	2013	KGI1	*Pararhyssocolpus paradoxus*; *Amblydorylaimus isokaryon*
**Livingston Island, Punta Hesperides (LI)**			
Grass spot (*Deschampsia antarctica* E. Desv.), on a high rock, on the beach near Johnson Dock inlet.	1994	DA	*Amblydorylaimus isokaryon*
A moss-grass (*Deschampsia antarctica*-*Polytrichum* sp.) community, on top of a small flat rock, on the beach near Johnson Dock inlet.	1994	DAP	*Amblydorylaimus isokaryon*
A small moss tuft (*Sanionia* sp.), transect over a large rock.	1994	S	*Pararhyssocolpus paradoxus*
Moss *Sanionia georgico-uncinata* (Müll. Hal.) and grass *Deschampsia antarctica*.	2001	HPPS	*Amblydorylaimus isokaryon*
Grasses *Colobanthus quitensis* (Kunth) Bartl. and *Deschampsia antarctica*, moss.	2003	CDM	*Pararhyssocolpus paradoxus*, *Amblydorylaimus isokaryon*
Grasses *Deschampsia antarctica*, *Colobanthus quitensis*.	2003	SDC	*Pararhyssocolpus paradoxus*; *Amblydorylaimus isokaryon*
Grass *Deschampsia antarctica*, moss.	2003	DM	*Pararhyssocolpus paradoxus*
**Nelson Island (NI)**			
*Duthoit Point* Moss	2013	M	*Pararhyssocolpus paradoxus*; *Amblydorylaimus isokaryon*

#### Measurements.

See Table 2.

**Table 2.** Morphometrics of *Amblydorylaimus isokaryon* (females and males). All measurements, unless indicated otherwise, are in µm (and in the form: mean±SD (range).

Locality	Nelson Island		Livingston Island									King George Island	
	M		SDC		HPPS		CDM		DA		DAP	KGI1	
Characters	♀(n=10)	♂ (n=10)	♀ (n=4)	♂	♀	♂	♀	♂	♀	♂	♂	♀	♂
L (mm)	2.85±0.25 (2.47–3.31)	2.88± 0.18 (2.58–3.24)	3.08±0.09 (3.00–3.20)	3.14, 3.23	3.00, 3.32	2.62, 3.00, 3.09	3.01, 3.02	2.76	2.63, 2.76, 2.89	2.96	2.76	2.13	2.65
a	29.2±2.6 (25.6–32.9)	31.6± 2.4 (28.5–34.8)	27.6±1.2 (26.6–29.3)	33.2, 29.6	35.3, 38.2	31.4, 34.1, 38.3	28, 27.3	30.3	27.3, 29.9, 29.6	35.2	36.7	26	34.2
b	4.1± 0.3 (3.7–4.4)	4.2± 0.2 (3.9–4.4)	4.4± 0.1 (4.3–4.5)	4.4, 4.6	4.6, 4.4	4.1, 4.1, 4.4	4.1, 4.3		3.6, 4.0, 4.0	4.2	4.3	3.8	4.8
c	70.6±7.4 (59.7–79.1)	66.6±5.1 (59.9–77.2)	73.7±2.1 (71.2–75.9)	74.8, 83.8	63.8, 77.7	62.0, 75.8, 69.2	73.4, 79.3	61.3	72.1, 57.6,79.3	64.0	65.6	68.6	62.4
c‘	0.9±0.1 (0.8–1.0)	0.9±0.1 (0.8–1.0)	1.0±0.1 (0.9–1.0)	1.0, 0.9	1.0, 1.0	0.9, 0.9, 1.0	0.9, 0.9	0.9	0.9, 1.0, 0.9	1.0	0.9	0.8	0.9
V %	52.2± 2.0 (50–56)		53.5± 1.3 (52–55)		54, 53		52, 52		54, 57, 58			53	
Lip region width	24.4±0.7 (23–25.5)	25.1±1.0 (23.5–27)	24.1±1.1 (23–25.5)	24, 26	25, 27	24.5, 24, 24	24, 24	25	25.5, 24, 26	25.5	23	24	26
Odontostyle	31.1±0.7 (30–32)	32.1±1.4 (30–35)	30.7±0.6 (30–31.5)	30, 30	31, 32	31, 30, 31	30, 30	29	30, 31, 32	30	29	29	29
Odontophore	51.9±2.0 (50–56)	52.03±3.4 (47.5–56)	53.4±1.3 (52–54)			-, 57, 51	54, -					46	
Pharynx	703.2±41.0 (632.5–756)	695.3±40.9 (650–765)	706.1±10.3 (692–716)	710, 707	652, 745	646, 732, 706.5	730, 700		737, 698, 715	697	648	566	550
Width at pharynx base	89.8± 8.4 (78–104)	87.3± 6.1 (75.5–94)	93.6±7.5 (83–99)	93, 98	81, 83.5	79, 83, 78	102, 105		87, 87, 94	83	73	75	73
Width at mid body	97.9±9.0 (85–110)	91.4±6.2 (79–101)	111.8±6.1 (103–115)	95, 109	85, 87	83, 88, 81	108, 111		96.5, 92, 98	84	75	82	77
Prerectum length	106.2±11.1 (92–131)	136.1±11.2 (120–149)	88.4±8.6 (82–101)	-, 154	110, 104.5	130, 154, -	100, 105		-, 117,131			58	
Rectum length	52.6±3.6 (46–58)		49.4±1.8 (47–51)		55.5, 48		54, 48		56.5, 62, 52			52	
Tail	40.5±3.6 (35–47)	43.4±1.6 (41–46)	41.9±2.3 (40–45)	42, 39	47, 43	42, 40, 45	41, 38	45	36.5, 48, 36.5	46	42	31	42
Spicules		95.1±3.3 (89–100)		91, 96.5		93, 93.5, 93		86		94	88		93
Ventromedian supplements		12–16		15, 12		15, 14, 13		14		15	15		14

#### Description.

*Female.* Body large, curved ventrad after fixation, especially in posterior end. Cuticle 2–4 µm thick at postlabial region, 4–6 µm at mid-body and on tail posterior to anus, three-layered, outer layer thin with fine and distinct transverse striation (especially well visible on SEM, annules 0.4–0.7 µm wide ); intermediate layer also thin, refractive, especially on tail region; inner layer thicker than the others. Lateral chord occupying 20–27% of midbody diam. Lateral pores well perceptible, often conspicuous throughout entire body, 10–14 in number in neck region, dorsal pores 3–4, ventral pores along the whole body, 9–11 in neck region. Lip region angular, set off from the adjoining body by a constriction; 2–3 times as wide as high, about 23–32% of body diameter at neck base. Based on SEM photographs oral aperture dorso-ventral, vestibulum hexagonal; labial and cephalic papillae prominent, labial papillae mamilliform, surrounded by circular annules, cephalic papillae button-like, without such annules, perioral field slightly elevated. Amphidial fovea cup shaped its aperture 41–52% of lip region diam., fusus (sensillium pouch) at 29–32 µm from anterior end, small posterior pouches present which are not always discernable. Odontostyle long, weakly sclerotised, 7–9 times as long as wide, 1.2–1.4 times lip region diam., aperture occupying 1/3 to more than 1/2 of its length (30–53%), av. 2/5, depending on the position of the body (under SEM it is seen that the aperture reaches 12–14 µm which is about 2/5 of the odontostyle length), two edges of the slit do not overlap. Guiding sheath distinct, its anterior edge located at 11–13 µm (or 0.4–0.6 times lip region diam. to anterior end (in not protruded odontostyle)) and seems cuticulised stronger than the posterior edge which is located at the base of odontostyle. Odontophore rod like, 1.5–1.9 times long as odontostyle. Anterior region of pharynx enlarging gradually, basal expansion 350–450 µm, (321 µm in the specimen from King George Island), occupying 50–60% of total neck length. Anterior subventral nuclei equal in size and shape, slightly smaller than dorsal nucleus; dorsal gland nucleus 4.5–6 µm diam., first and second pair subventral gland nuclei 4–4.5 and 3–4 µm diam., respectively. Location of pharyngeal glands and their orifices is presented in Table 3. Cardia rounded conoid, ending with sharply pointed tongue, variable in size and shape. In some specimens a dorsal cellular mass present at cardia level. The posterior end of the intestine with tongue-like structure of variable length. Prerectum 1.9–3.2 (1.5 times in specimen from King George Island), rectum 1.0–1.5 times anal body diam. long. Prerectum separated from intestine by a transverse muscular ring. Sphincter between rectum and prerectum well developed. Female genital system didelphic amphidelphic, both branches equally and well developed – anterior 510.1±86.5 (390–695.5) µm and posterior 527.4±67.9 (430–705.5) µm long, respectively (anterior 313 and posterior 347 µm long in specimen from King George Island). Ovaries short well developed, often not reaching sphincter level. Oviduct with well-developed *pars dilatata*, often containing sperm. Sphincter between *pars dilatata oviductus* and uterus small, well developed. Uterus long, anterior 232–435 µm, posterior 226–395 µm long, without differentiation, filled with sperm. Vulva longitudinal (based on SEM observations, Fig. 12A, B). Vagina extending inwards for 51–68% of body diam.; *pars proximalis* 36–44×15.5–23 µm, *pars refringens* with two trapezoidal sclerotisations, with combined width of 17.5–22 µm; *pars distalis* 6–10 µm long. Three females each with one uterine egg, measuring 123–135×56–82.5 µm, in one female one egg located in *pars dilatata oviductus*, measuring 123×80 µm. Irregularities of body cuticle present around vulva, on SEM observation they appeared as additional cuticle masses. Tail short conoid, ventrally arcuate, with bluntly rounded tip, 1.3–1.7% of body length. Caudal pores two pairs, on SEM appeared papillae-like.

**Table 3.** Pharyngeal characters of *Amblydorylaimus isokaryon*. For abbreviations see Loof and Coomans (1970) and Andrássy (1998b).

Locality	Nelson Island				Livingston Island				King George Island	
		M			SDC		CDM		KGI1	
Characters	n	female	n	male	female	male	female	male	female	male
DO	4	51–53	2	45, 53		53	51		48	51
DN=D	7	54–57	6	54–57	58, 59	58	57, 55	60	54	56
Distance DO-DN %	4	3–5	2	5–10		5	6		6	9
S_1_O_1_	4	70–74	2	65, -		71	70		70	69
S_1_O_2_	3	75–77	2	71.5, 75		76	76		75	75
S_1_N_1_	7	69–72	6	69–72	72, 73		71, 70	73	69	70
S_1_N_2_	7	74.5–76	6	73–76	77		77	79	74	75
S_2_O	4	87–88	2	86, 88.5		88	88		87	87
S_2_N_1_	7	84–85.5	6	83–86	87, 86	86	85, 85	87	84	
S_2_N_2_	7	84–87	6	84–86	87, 86	87	87, 85	87	85	84
AS_1_	7	30–38	6	31–35	33, 35		33, 33	33	33	31
AS_2_	7	42–45	6	40–45	45		46	47	43.5	43
PS_1_	7	63–68	6	62–68	69, 66	68	66, 66	67	66	
PS_2_	7	63–70	6	64–68	69,67	68	69, 68	67	67	64

*Male.* General morphology similar to that of the female, except for the genital system. In one specimen the odontostyle aperture ventral. Arrangement of pharyngeal gland nuclei and their orifices is presented at Table 3. Genital system diorchic, with opposite testes: anterior 376.4±28.9 (339–418) µm and posterior 361.9±56.8 (279–442) µm long (n=6), (anterior 237 and posterior 255 µm in a specimen from King George Island), respectively. Spicules dorylaimoid, strongly curved ventrad and robust, their length about 1.7–2.3 times cloacal body diam. Ventromedian supplements preceded by one adcloacal pair of papillae, 12–16 in number, regularly spaced, with small cuticular folds between them, adcloacal pair located at 29–37.5 µm apart from cloacal opening (26 µm in specimen from King George Island). Sperm spindle shaped, measuring 11–13×3–4 µm. Lateral guiding pieces, cylindrical with bifurcate end, measuring 24–31×3–5 µm. Tail short conoid, ventrally arcuate, with obtusely rounded tip, two pairs of caudal pores.

*Juveniles.* Morphometrics obtained from juvenile specimens, and the relationship between the lengths of their functional and replacement odontostyles and body lengths, identified four juvenile stages (Figure 13). Tail in J1-J3 elongated conoid, ventrally arcuate with rounded terminus, in J4 as in females, c’ decreasing during successive stages to female (Table 4, Figs 6, 10).

**Figure 1.**
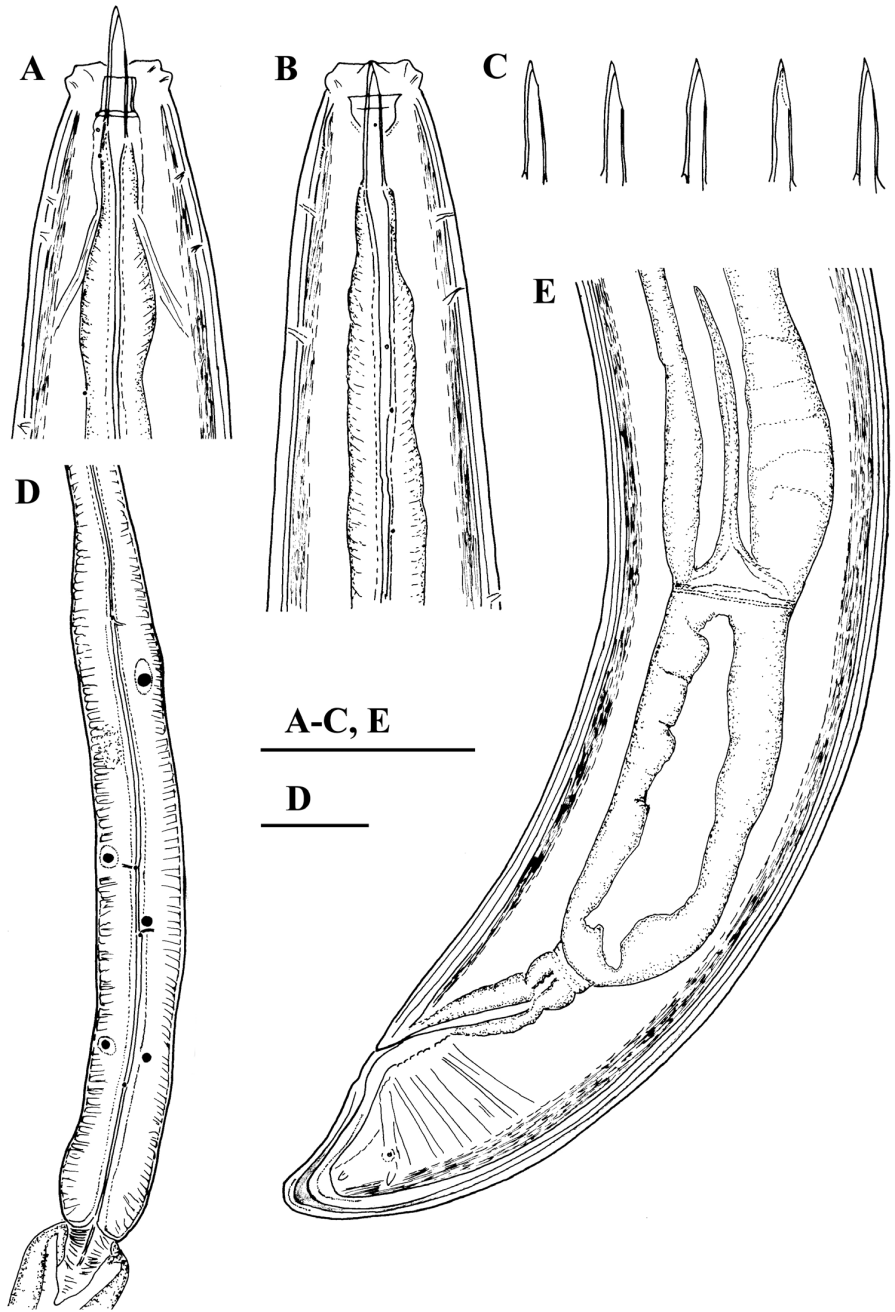
*Amblydorylaimus isokaryon* (Loof, 1975). *Female*: **A, B** Anterior ends (**A** NI; **B** LI, SDC) **C** Odontostyle variations **D** Pharyngeal expansion, pharyngeal glands, cardia (KGI) **E** Posterior end (NI). Scale bar: 50 µm (**A–E**).

**Figure 2.**
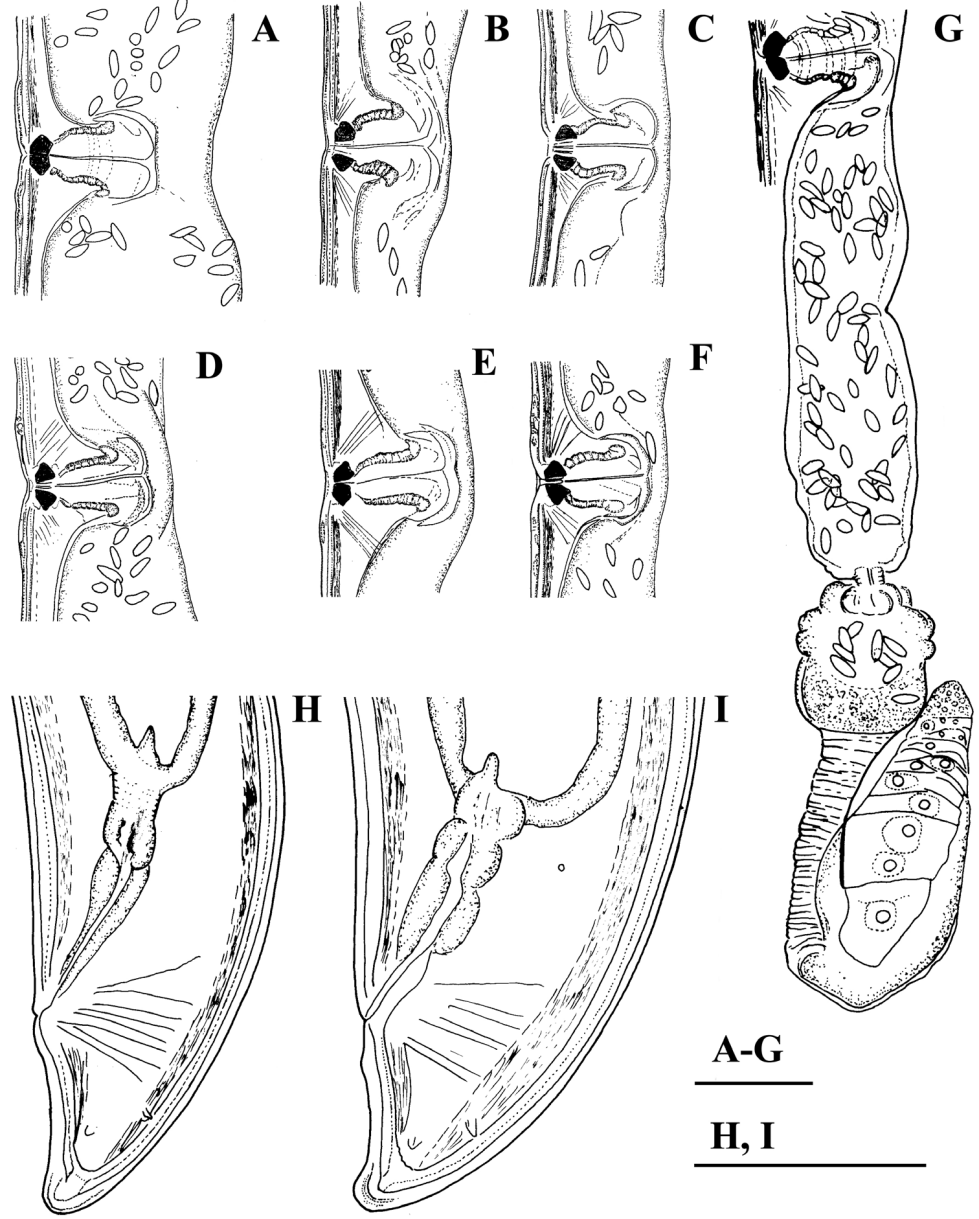
*Amblydorylaimus isokaryon* (Loof, 1975). *Female*: **A–F** Vulval region (**A** LI, SDC; **B–F** NI) **G** Posterior genital branch (LI, CDM) **H, I** Tail ends (NI). Scale bar: 50 µm (**A–I**).

**Figure 3.**
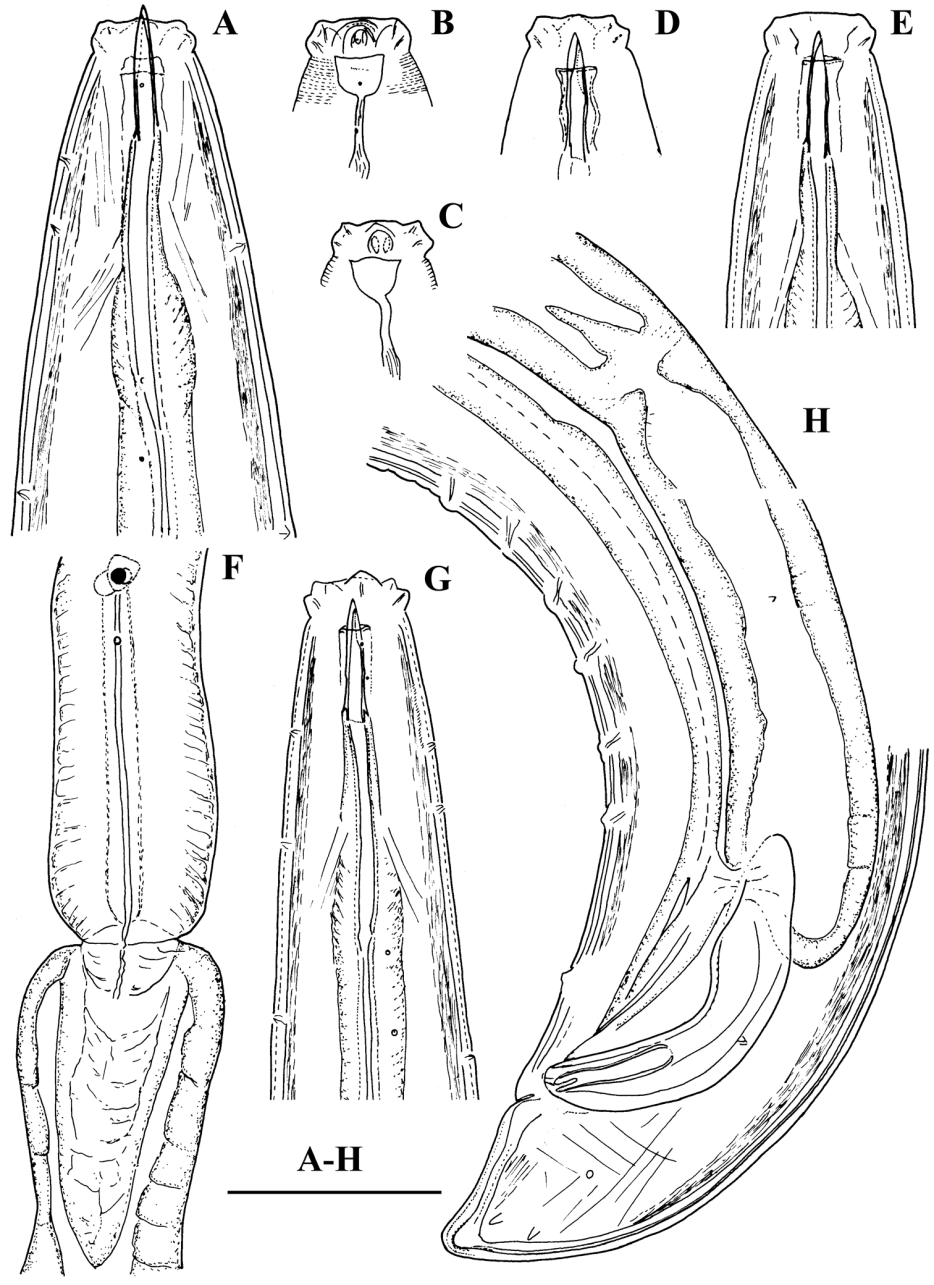
*Amblydorylaimus isokaryon* (Loof, 1975). *Male*: **A, D, E, G** Anterior end (**A** NI; **D, E, G** LI, HPPS) **B, C** Amphidial fovea (**B** NI; **C** LI, HPPS) **F** Posterior ventrosublateral glands, cardia (KGI) **H** Posterior end (NI). Scale bar: 50 µm (**A–H**).

**Figure 4.**
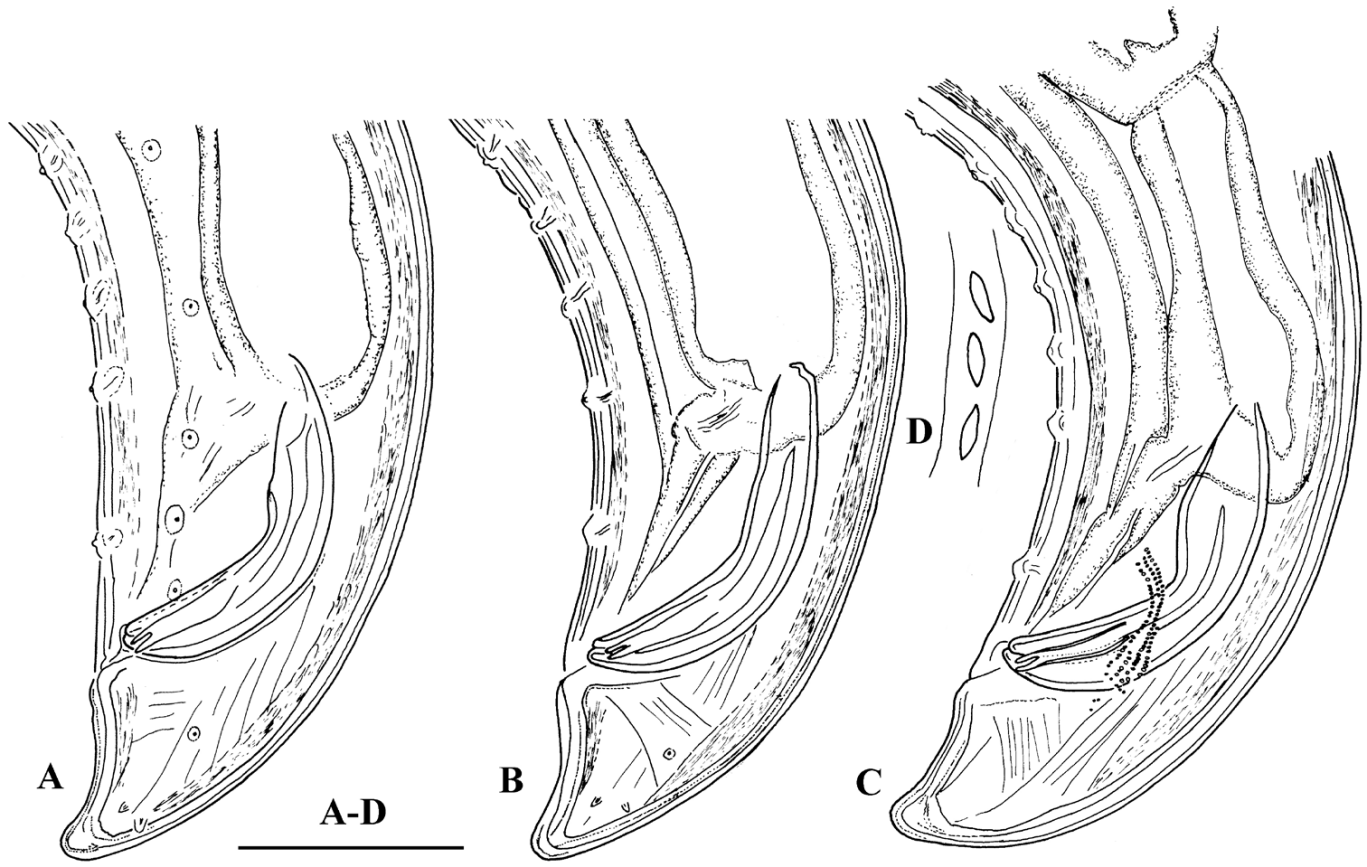
*Amblydorylaimus isokaryon* (Loof, 1975). *Male*: **A–C** Posterior ends (**A** LI, HPPS; **B** NI; **C** KGI) **D** Sperm in *ductus ejaculatoris* (**KGI**). Scale bar: 50 µm (**A–D**).

**Figure 5.**
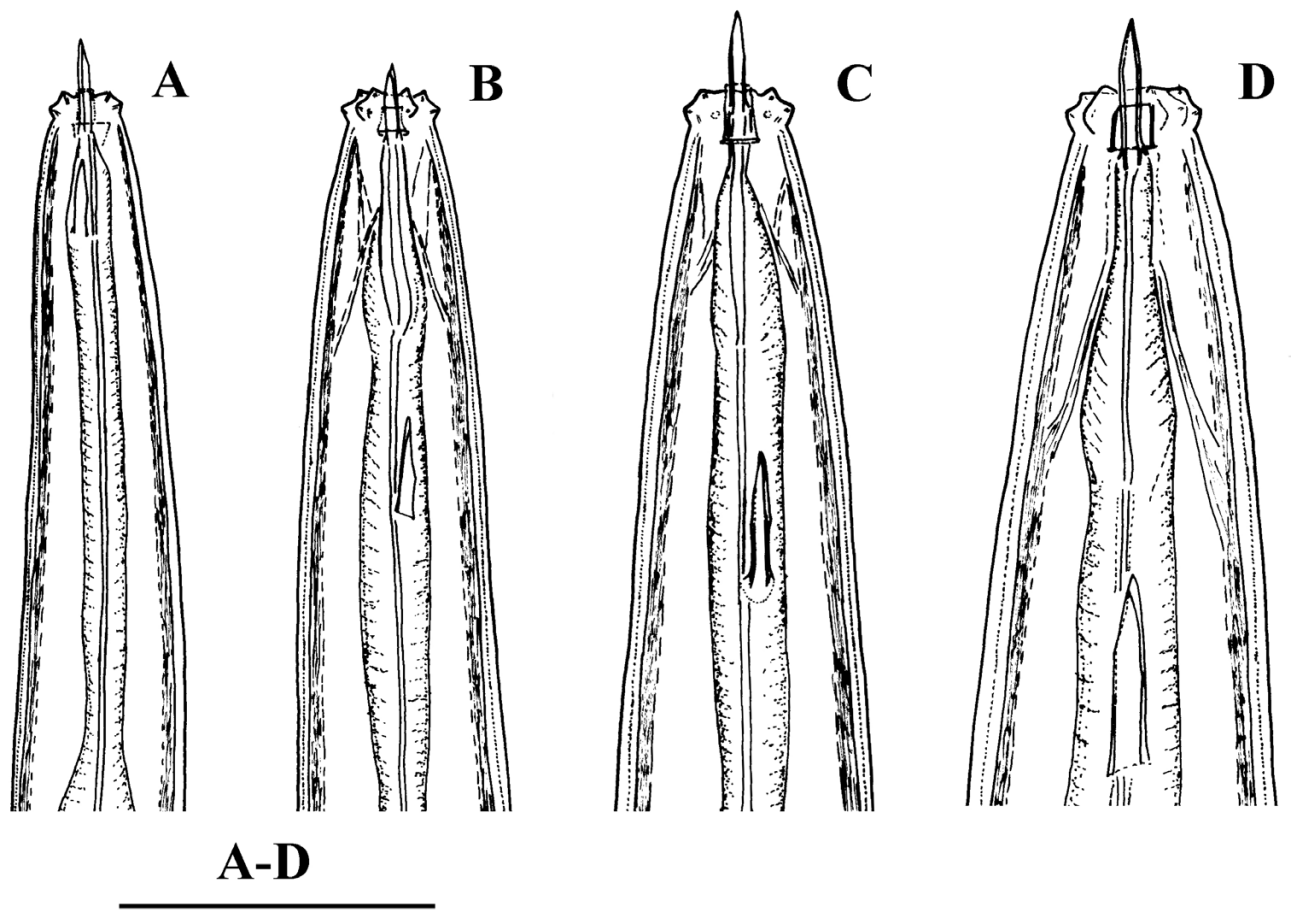
*Amblydorylaimus isokaryon* (Loof, 1975). *Juveniles*: **A–D** Lip region of J1-J4 (**NI**). Scale bar: 50 µm (**A–D**).

**Figure 6.**
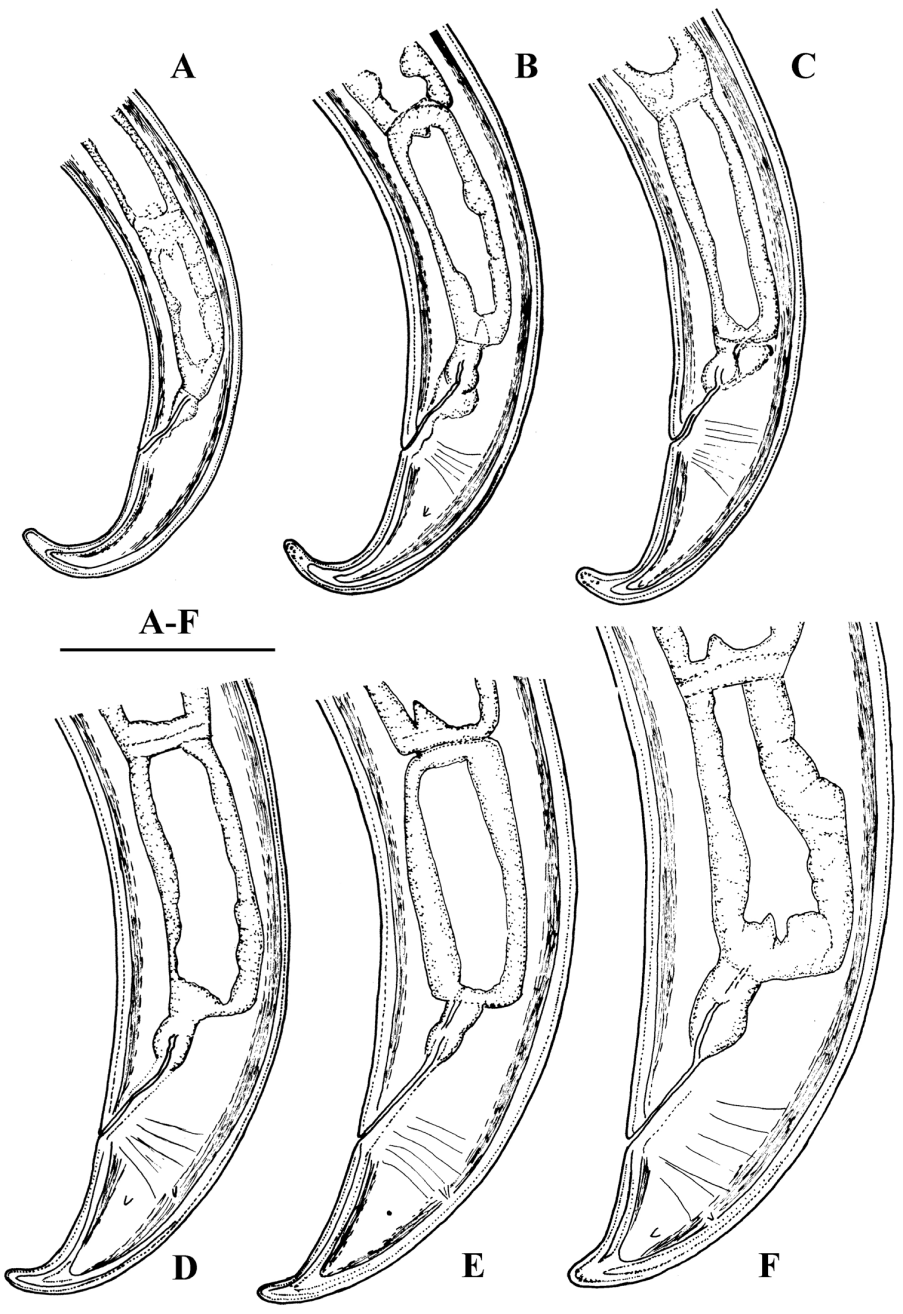
*Amblydorylaimus isokaryon* (Loof, 1975). *Juveniles*: **A–F** Tail ends (**NI**) **A** J1 **B, C** J2 **D, E** J3 **F** J4. Scale bar: 50 µm (**A–F**).

**Figure 7.**
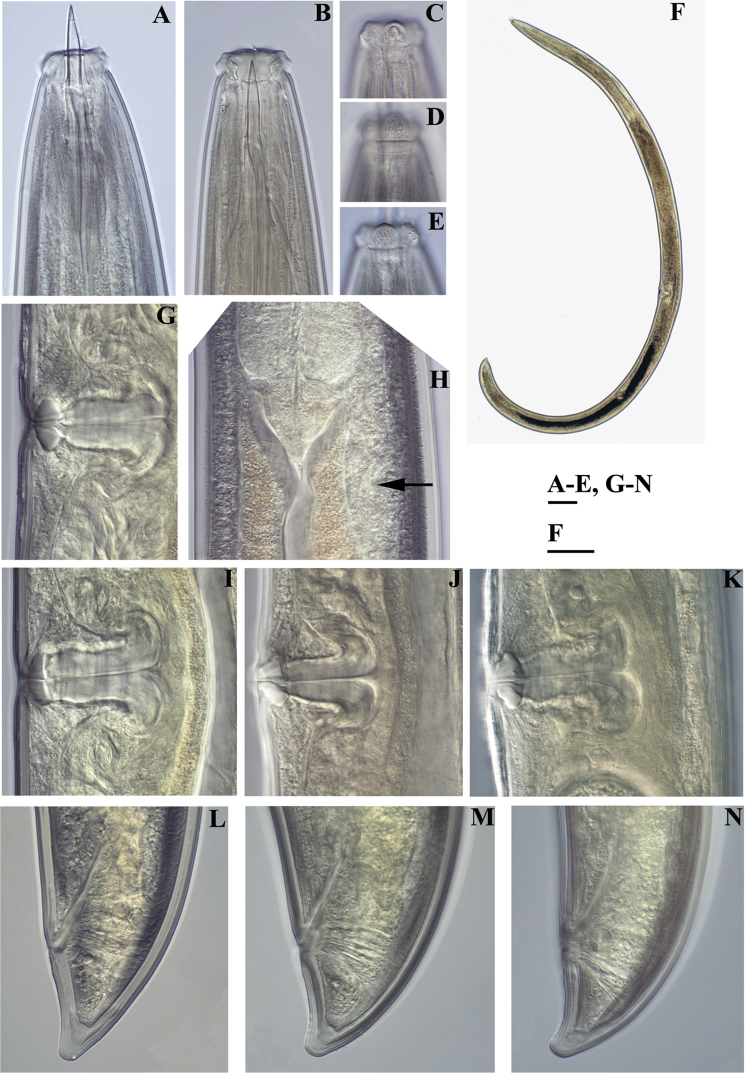
*Amblydorylaimus isokaryon* (Loof, 1975). *Female*: **A, B** Anterior ends (**A** NI; **B** LI, HPPS) **C–E** Amphidial fovea (**C** LI, HPPS; **D, E** NI) **F** Entire body (**NI**) **G, I–K** Vulval region (**G, I** NI; **J, K** LI, HPPS) **H** Cardia and dorsal cellular mass (marked by an arrow) (NI) **L–N** Tail ends (**L, M** NI; **N** LI, HPPS). Scale bars: 10 µm (**A–E, G–N**); 200 µm (**F**).

**Figure 8.**
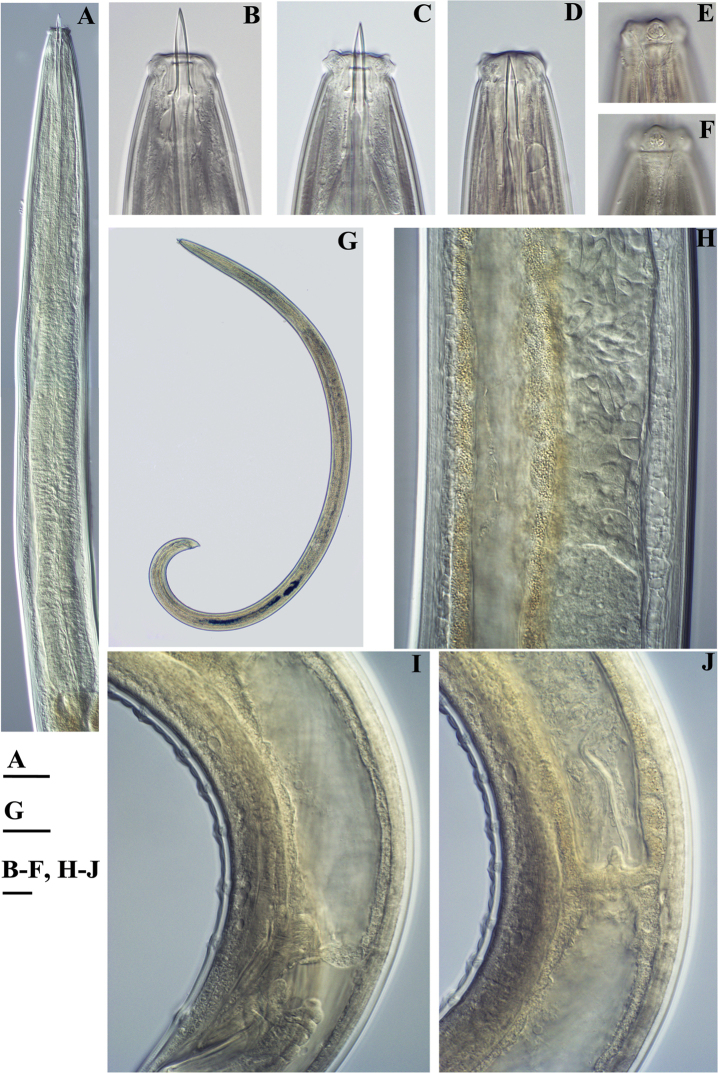
*Amblydorylaimus isokaryon* (Loof, 1975). *Male*: **A** Pharyngeal region (NI) **B–D** Lip region (**B, C** NI; **D** LI, HPPS) **E, F** Amphidial fovea (**E** LI, HPPS; **F** NI) **G** Entire body (NI) **H** Part of testis with sperm (NI) **I** Prerectum, rectum and ejaculatory glands (LI, HPPS) **J** Tongue-like projection (LI, HPPS). Scale bars: 50 µm (**A**); 10 µm (**B–F, H–J**); 200 µm (**G**).

**Figure 9.**
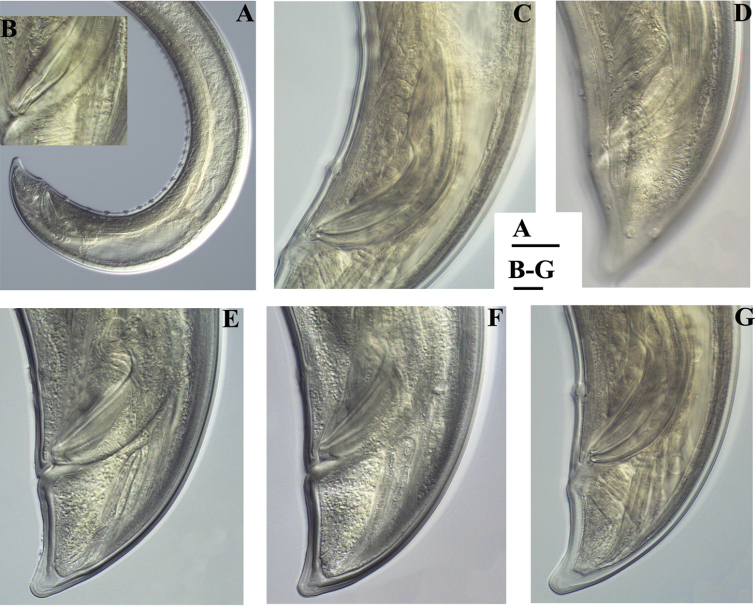
*Amblydorylaimus isokaryon* (Loof, 1975). *Male*: **A** Posterior end (LI, HPPS) **B** Lateral guiding piece (NI) **C** Spicula (LI, HPPS) **D** Tail end, caudal pores (NI) **E–G** Tail ends (**E, F** NI; **G** LI, HPPS). Scale bars: 50 µm (**A**); 10 µm (**B–G**).

**Figure 10.**
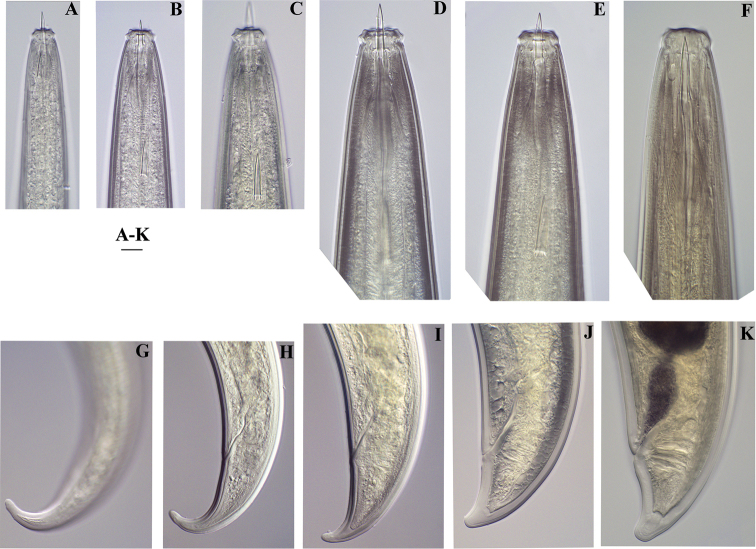
*Amblydorylaimus isokaryon* (Loof, 1975). **A–E, G–J** *Juveniles* (NI): **A–E** Lip region of **A** J1 **B** J2 **C** J3 **D, E** J4 **G–J** Tail ends J1-J4 **F, K** *Female* (LI, HPPS): **F** Anterior end **K** Tail end. Scale bar: 10 µm (**A–K**).

**Figure 11.**
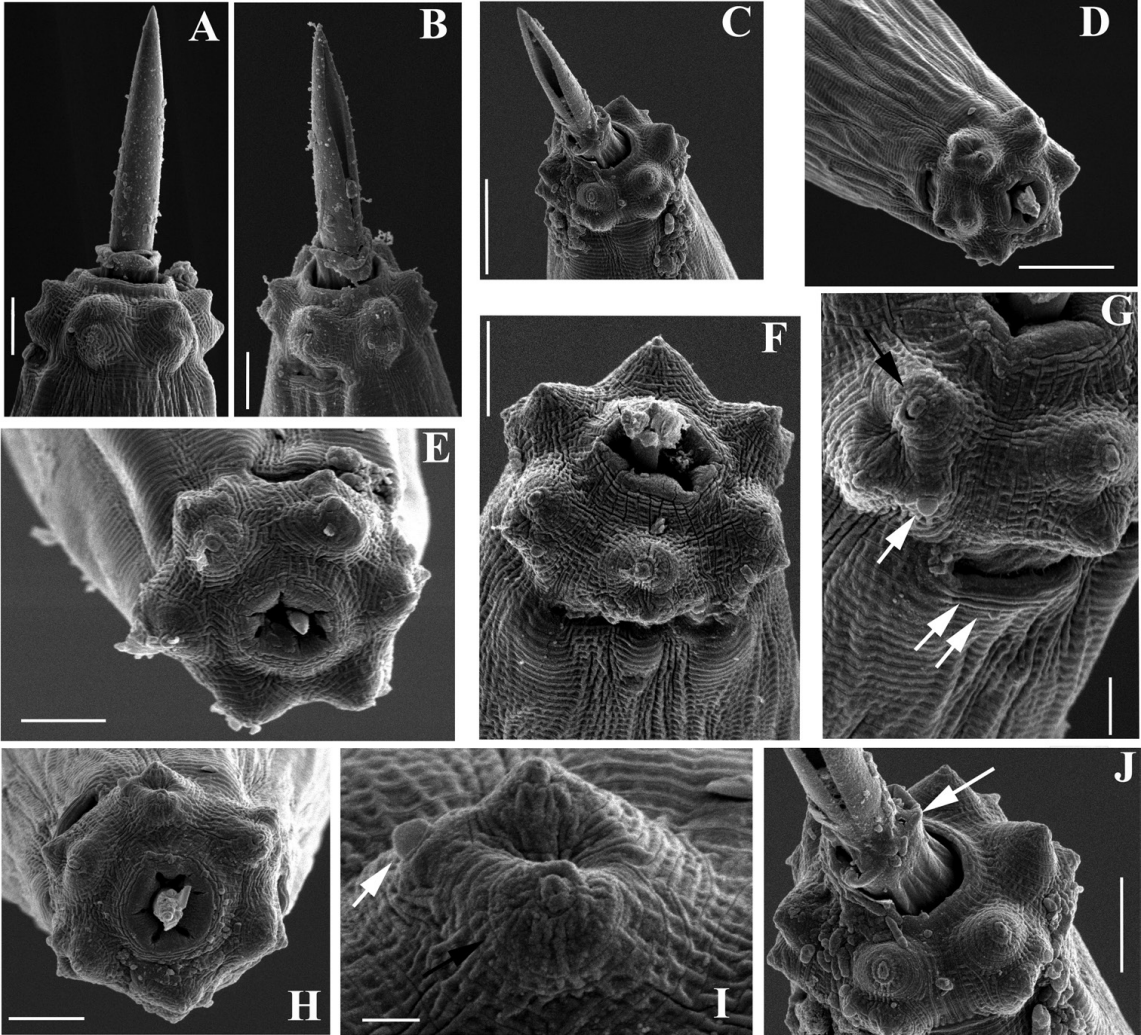
SEM micrographs. *Amblydorylaimus isokaryon* (Loof, 1975). *Female* (**NI**): **A** Lip region with protruded odontostyle **D, E** Lip region, in face view **G, I** Cephalic (marked by white arrow) and labial papillae (marked by black arrow), amphid aperture (marked by two arrows) *Male*: **B, C** Lip region, odontostyle aperture **F, H** Lip region **J** Cephalic and labial papillae, anterior edge of guiding sheath (marked by an arrow). Scale bars: 5 µm (**A, B, E, F, H, J**); 10 µm (**C, D**); 2 µm (**G**); 1 µm (**I**).

**Figure 12.**
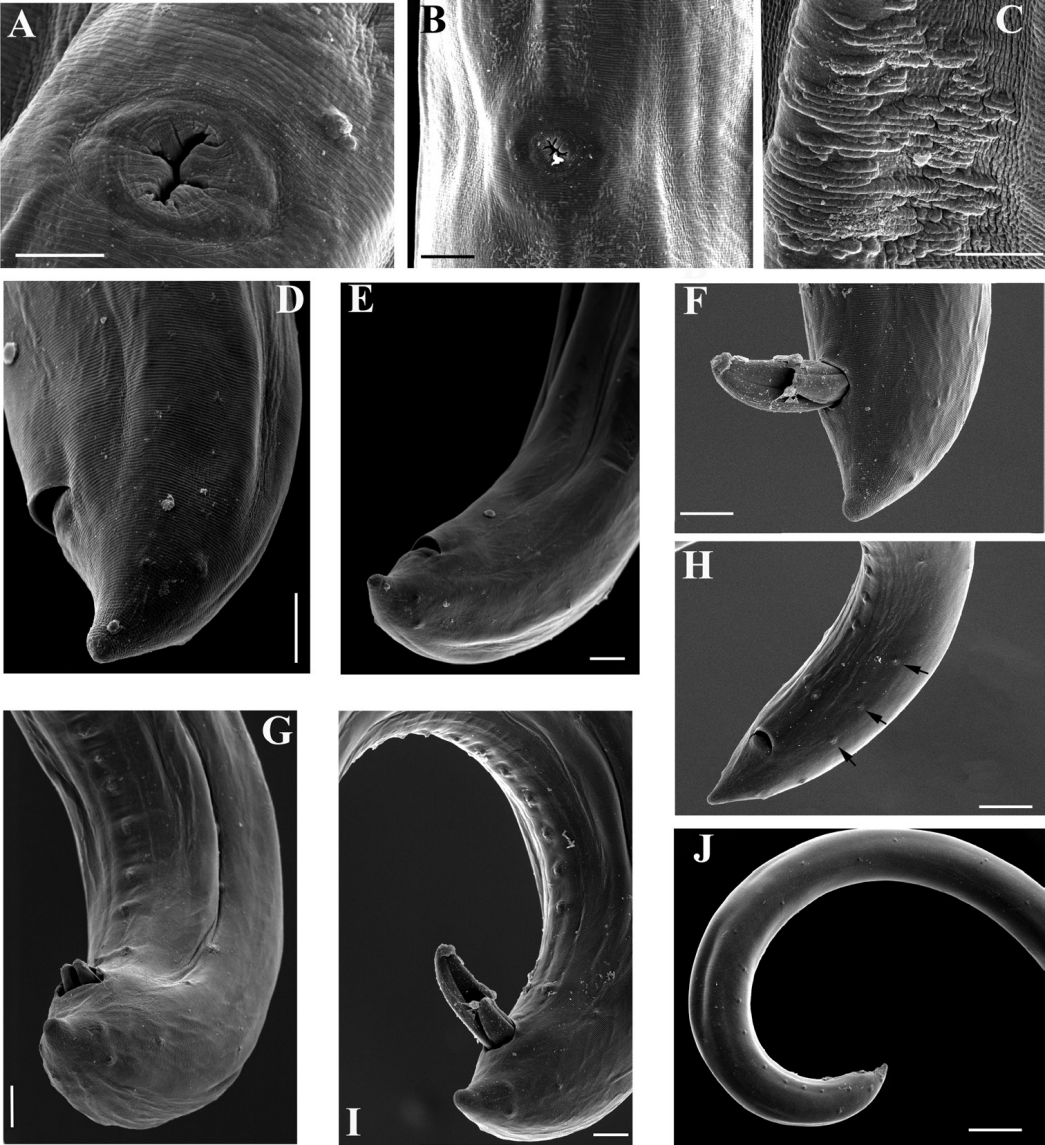
SEM micrographs. *Amblydorylaimus isokaryon* (Loof, 1975). **A–E** *Female*: **A, B** Vulval region (NI) **C** Irregularities around vulva (LI) **D, E** Tail ends (NI) **F–J** *Male* (NI): **F** Tail end with protruded spicules **G** Tail end (ventral view), spicules, ventromedian supplements **H** Posterior end, ventromedian supplements and lateral pores (marked by arrows) **I, J** Posterior ends. Scale bars: 5 µm (**A, C**); 10 µm (**B, D–H**); 50 µm (**J**); 20 µm (**I**).

**Figure 13.**
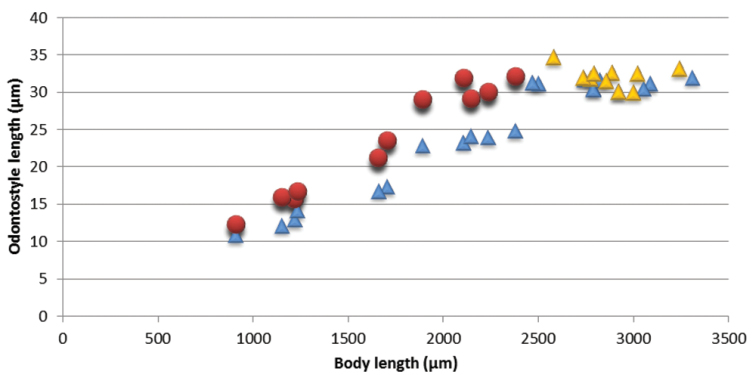
*Amblydorylaimus isokaryon* (Loof, 1975). Scatter plot of the functional (●) and replacement odontostyle (▲) in relation to the body length of the juvenile stages and adults: females (▲) and males (▲).

**Table 4.** Morphometrics of *Amblydorylaimus isokaryon* (juveniles). All measurements, unless indicated otherwise, are in µm (and in the form: mean±SD (range).

Locality	Nelson Island				Livingston Island	
	M				DA	DAP
Characters/Stages	J1	J2	J3	J4 (n=5)	J4	J4
L (mm)	0.91	1.22, 1.15, 1.23	1.70, 1.66	2.15±0.2 (1.89–2.38)	2.53	2.43
a	30.2	29.9, 26.5, 29.6	27.7, 28.6	27.9±2.1 (24.9–30.2)	34.5	36.2
b	3.5	3.6, 3.4, 3.4	3.9, 4.0	4.0±0.2 (3.8–4.4)	4.1	4.1
c	18.0	22.7, 21.7, 24.4	35.4, 34	54.8±4.3 (49.6–59.5)	57.8	65.6
c‘	2.5	2.4, 2.1, 2.1	1.6, 1.7	1.1±0.1 (1.1–1.2)	1.3	1.2
Lip region width	11	14, 14, 14	16.5, 16	19.9±1.4 (18–22)	19	20
Odontostyle	11	13, 12, 14	17, 17	23.9±0.8 (23–25)	22	24
Replacement odontostyle	12	16, 16, 17	24, 21	30.5±1.5 (29–32)	29	29
Pharynx	262.5	338, 342, 359	438, 420	538.0±41.3 (485.5–595)	612	592.5
Width at pharynx base		40.5, 40, 41	59, 52	71.4±5.4 (65–78)	68	65
Width at mid body	30	41, 43, 42	61, 58	77.2±7.2 (69.5–85)	73	67
Prerectum length		58.5, 59, -	65, 67	80.7±13.1 (68–97)	67	91
Rectum length		25, 24, 23.5	37, 29	41.1±1.4 (39–42)	45.5	43
Tail	50	53.5, 53, 50.5	48, 49	39.4±3.7 (35–43.5)	44	37
Genital primordium	17	-, 23, -	25, 25			

#### Sequence and phylogenetic analyses.

The BLAST search using D2-D3 region sequence of *Amblydorylaimus isokaryon* showed highest similarity (96%) to *Aporcelaimellus salicinus* Álvarez-Ortega, Subbotin & Peña-Santiago, 2013 (JX094341–42), while the 18S rDNA sequence was closest (99% similarity, 4–10 nucleotide differences of about 1700 bp) to several *Aporcelaimellus* spp., *Allodorylaimus andrassyi* (Meyl, 1955) Andrássy, 1986 and four sequences acquired during environmental studies of arable soil (Griffiths et al. 2006) and trembling aspen rhizosphere (Lesaulnier et al. 2008). Since the 18S rDNA phylogram based on bigger dataset (Figure 24) did not show clearly the evolutionary relationships of *Amblydorylaimus isokaryon* a smaller dataset with the closest sequences was analysed (Figure 25). Although the very low 18S rDNA resolution this analysis yielded a tree with *Amblydorylaimus isokaryon* being a part of well-resolved group of species assigned to three families: Dorylaimidae De Man, 1876 (*Labronema vulvapapillatum* (Meyl, 1954) Loof & Grootaert, 1981 and *Mesodorylaimus centrocercus* (de Man, 1880), Geraert, 1966, Qudsianematidae (*Ecumenicus* spp.) and Aporcelaimidae (includes mainly *Aporcelaimellus* spp. and *Allodorylaimus andrassyi* which probably is misidentified). Further, in the 28S rDNA-based phylogenetic tree *Amblydorylaimus isokaryon* appeared a sister species of *Aporcelaimellus salicinus*, again being a part of a well-supported group of several *Aporcelaimellus* spp. and *Allodorylaimus andrassyi* (Figure 26).

#### Discussion.

The main morphological characters of the studied populations are very similar, only the specimen from King George Island differs by its shorter body, pharynx, pharyngeal expansion, anterior and posterior female genital branches, prerectum and tail (Table 2). Our materials generally agree well with the type specimens (Loof, 1975), although some differences occurred: the present specimens have broader lip region (length of odontostyle 1.2–1.3 *vs* 1.5 times longer than lip region diam., longer uterine eggs (123–135 *vs* 117–122 µm), and somewhat longer distance adcloacal pair of papillae – cloaca (29–37.5 *vs* 26–29 µm) (Andrássy 1998a). Loof (1975) described the vulva as longitudinal but according to Andrássy (1998a) it is more or less a roundish pore, although he may not have observed females in ventral position. Our SEM studies confirm Loof’s observations. Andrássy (1998a) reported that spermatozoids have an atypical shape for dorylaimids being rounded or potato-like, however our observations showed that their shape is spindle-like, similar to the drawings by Loof (1975). Further, the presence of a tongue-like projection between the intestine and prerectum not mentioned in the original description was observed. None of the above mentioned authors reported the cuticular irregularities around the vulva documented here both by LM and SEM.

This species was originally described as *Eudorylaimus isokaryon* by Loof (1975); later Andrássy (1998a) established a new genus, *Amblydorylaimus* to accommodate it on the basis of several morphological characters (amphidial fovea and odontostyle shape, equally sized mid-pharyngeal nuclei, atypical sperm shape, nipper-like adspicular pieces and unusual location of adcloacal pair of supplements). He described and illustrated *Amblydorylaimus isokaryon* having a specific shape of odontostyle – resembling garden shears; the aperture appeared small. He suggested that this unusual shape was not caused by fixation artefacts as “other organellum of cuticular origin is clearly visible, without any deformation” and “all other *Eudorylaimus* species collected by Spaull (Loof, 1975) in his study trip do possess normally shaped, well preserved dorylaimid spear”. Andrássy (1998a) suggested that it would be necessary to know if living specimens possessed this shape of odontostyle. We examined living specimens of this species, and did not find the peculiarities of the odontostyle shape observed by this author. In the original description, Loof (1975) did not mention this special feature of odontostyle and noted that the odontostyle aperture occupied one-third of its length. In our specimens the odontostyle is weakly sclerotised, regular with usual dorylaimid shape; the length of aperture longer, occupying 1/3–1/2 of the odontostyle. In earlier prepared slides, the odontostyle showed some irregularities similar to those described by Andrássy (1998a). The same author (1998a, 2009a) considered this genus as a member of family Qudsianematidae, but noted that it significantly differs from every genus of this family with its characteristic morphology. Molecular data based on 28S rDNA, however showed that this genus is a member of family Aporcelaimidae and not family Qudsianematidae. This conclusion is supported by our morphological evidences: large aperture of odontostyle (reaching almost ½ of odontostyle length), oral opening a dorso-ventral slit, cuticle thick with refractive layer, not fixed guiding ring etc. which confirm *Amblydorylaimus* fits better to the family Aporcelaimidae. Based on morphology and molecular data (28S) *Amblydorylaimus* is closely related to genus *Aporcelaimellus* Heyns, 1965 from which it can be differentiated by its longer and not robust odontostyle with shorter aperture (av. 2/5 *vs* more than 1/2 of odontostyle length), and not overlapping *vs* overlapping edges, lip region with radial *vs* bilateral symmetry (Álvarez-Ortega and Peña-Santiago 2013), vulva longitudinal *vs* transverse (except *Aporcelaimellus macropunctatus* (Heyns, 1967) Jimenez-Guirado, 1994 distinguished by its longitudinal vulva), position of adcloacal pair of papillae in males (more distant from cloacal opening *vs* very close) and lateral guiding pieces bifurcate *vs* simple. Recently, Andrássy (2009b) proposed a new genus close to *Aporcelaimellus* and *Amblydorylaimus*, the genus *Aporcelinus* Andrássy, 2009. The latter genus differs from the genus *Amblydorylaimus* by the structure of cardia (with a small dorsal lobe), transverse vulva, eggshell wrinkled, ventromedian supplements small, irregularly spaced, without precloacal space, location of adcloacal pair and shape of tail (conoid tail with sharply pointed terminus). Vinciguerra et al. (2014) believed that the taxonomic position of *Aporcelinus* is ambiguous; they noted that this genus could also be assigned to family Qudsianematidae on the basis of its morphological features (odontostyle aperture length, simple guiding ring and thickness of cuticle, composed of two layers). Related to the cuticle structure, it should be mentioned that genus *Aporcelinus* has three layered cuticle with inner refractive layer, well visible on several photomicrographs (Figs 4E, 8A–C) presented by Vinciguerra et al. (2014). Further, the location of adcloacal pair of male ventromedian papillae (comparatively far from cloaca opening) in *Amblydorylaimus isokaryon* shows some similarity to *Crassolabium persicum* Jabbari, Niknam, Vinciguerra, Moslehi, Abolafia & Peña-Santiago, 2012, but the latter species differs from it by the odontostyle structure (weakly sclerotised *vs* quite robust), not differentiated *vs* bipartite uterus, structure of *pars distalis* (without differentiation *vs* with two small sclerotisations close to the *pars refringens* in *Crassolabium persicum*) (Jabbari et al. 2012).

On the basis of morphological and molecular data, we propose the genus *Amblydorylaimus* to be transferred from family Qudsianematidae to the family Aporcelaimidae. It is worth mentioning that the latter family obviously is non monophyletic and we propose this taxonomic change on the base of the close relationships with the genus *Aporcelaimellus* now regarded as a member of family Aporcelaimidae.

#### Diagnosis (emended).

*Amblydorylaimus*.

#### Aporcelaimidae.

**Aporcelaiminae.** Body large, about 3 mm. Cuticle three-layered, outer layer thin with fine but distinct transverse striation. Lip region angular, offset from adjacent body by a constriction. Oral aperture dorso-ventral, hexagonal. Amphidial fovea caliciform with small posterior pouches. Odontostyle long, weakly sclerotised. Guiding sheath distinct, anterior and posterior edges moderately cuticularised. Odontophore rod like. Pharynx expanded in its posterior half. Nuclei distinct, dorsal nucleus fairly posterior in position, first subventral pair large and equal in size, posterior pair rather far from the end of pharyngeal expansion. Prerectum sharply separated from mid-intestine. Female genital system didelphic amphidelphic. Ovaries very short, uterus long without differentiation. Vulva longitudinal, cuticular irregularities present around it. *Pars refringens vaginae* well developed. The posterior end of the intestine with tongue-like structure. Sperm spindle shaped. Spicula dorylaimid, lateral guiding piece distally bifurcate. Ventromedian supplements numerous, regularly spaced, preceded by one adcloacal pair of papillae comparatively far from cloacal aperture. Tail similar in both sexes, short conoid, ventrally arcuate, with bluntly rounded tip. Tail in J1-J3 conoid elongated, in J4 as in female.

#### Distribution.

*Amblydorylaimus* is an endemic genus of the maritime Antarctic. It has been reported from several islands (Intercurrence, Elephant, Galindez, Livingston and King George) (Loof 1975; Maslen 1979; Peneva et al. 2009; Kito 2009). The present finding from Nelson Island represents a new geographical record.

### Pararhyssocolpus paradoxus

Taxon classificationAnimaliaDorylaimidaPararhyssocolpidae

(Loof, 1975) gen. n., comb. n.

Figures 14 , 15 , 16 , 17 , 18 , 19 , 20 , 21 , 22 , 23 , 24 , 25 , 26

Eudorylaimus paradoxus Loof, 1975
Rhyssocolpus paradoxus (Loof, 1975) Andrássy, 1986

#### Material examined.

Eighteen females, seven males and ten juveniles (J1, J3, J4) collected from three islands in Maritime Antarctic (Table 1).

#### Measurements.

See Table 5.

**Table 5.** Morphometrics of *Pararhyssocolpus paradoxus* n.gen., n.comb. (adults, juveniles). All measurements, unless indicated otherwise, are in µm (and in the form: mean±SD (range).

Locality	King George Island		Livingston Island						Nelson Island	King George Island		
Characters	KGI1		CDM		S	SDC	DM		M	KGI1		
n	♀(n=8)	♂	♀	♂	♀	♀	♀ (n=4)	♂	♀	J1	J3	J4 (n=7)
L (mm)	2.42±0.1 (2.18–2.69)	2.28, 2.40, 2.24	2.61	2.61	2.07, 2.36, 1.69	2.41	2.7±0.1 (2.35–2.87)	2.16, 1.96, 2.59	2.71	0.87	1.39, 1.37	1.93±0.2 (1.69–2.11)
a	23.4±0.98 (22.2–24.9)	22.8, 26.5, 22.8	21.6	22.1	20.6, 19.6, 18.0	23.1	22.6±1.2 (21.3–23.6)	21.3, 20.2, 23.4	24.3	26.6	24.2, 25.0	27.0±3.0 (22.5–32.0)
b	5.2± 0.4 (4.8–5.9)	5.2, 5.2, 4.9	5.7	5.9	4.7, 5.7, 4.5	5.8	5.7± 0.4 (5.3–6.2)	4.9, 4.4, 6.0	5.8	3.7	4.1, 3.9	4.8± 0.4 (4.1–5.2)
c	34.9±4.4 (28–40.4)	45.7, 34.7, 39.4	36.9	39.8	31.6, 31.6, 30.1	36.2	37.4±2.1 (35.6–40)	32.6, 30, 39.2	40.9	10.1	18.3, 15.8	24.4±3.8 (20.8–31.9)
c‘	1.5±0.2 (1.4–1.8)	1.2,1.2, 1.2	1.4	1.0	1.5, 1.6, 1.5	1.5	1.5±0.02 (1.4–1.5)	1.2, 1.2, 1.3	1.4	3.8	2.5, 2.4	1.9±0.2 (1.5–2.2)
V %	46.6± 1.5 (44–49)	-	49	-	46.5, 47, 48	49	47.6± 2.2 (44–49)	-	47	-	-	-
Lip region width	20.7±0.9 (20–22.5)	20, 21.5, 21	22	22	21, 22, 21.5	20.3	21.4±0.5 (21–22)	22, 21, 21	22	10	15, 16	18.7±0.5 (18–20)
Odontostyle	19.8±0.6 (19–21)	19, 19, 20	20	20	20.5, 21, 19	21	20.5±0.5 (20–21)	20, 21, 19	20	10	14, 13	17±1.0 (16–18)
Replacement odontostyle	-	-	-	-		-	-	-	-	11	17, 16	19.9±0.9 (18–21)
Odontophore	41.1±1.9 (39–43)	40	45	-		44	43, 43	-, 45, 37	-	-	-	-
Anterior end guiding ring	16	-	15	15	14, 15, -	-	15	15, 15.5, -	-	-	-	-
Anterior end nerve ring	162.9±9.3 (151–177.5)	160, 162.5, 152.5	177.5	-	-	160	168.3±10.1 (154–178)	172, 176, 151	-	-	-	-
Pharynx	463.9±10.1 (456–487)	443, 461, 462.5	460	441	444, 414, 375	417	470.2±21.4 (446–494)	444, 447, 431	470	237	344, 356	405.4±12.6 (382–418)
Width at pharynx base	88.3± 6.8 (78–97)	88, 81, 84.5,	103	108	93, 99, 81	94.5	91.8± 7.9 (93–110.4)	92.5, 86, 94	97	-	-	-
Width at mid body	103.7±9.8 (88.5–115)	100, 91, 98	121	118	101, 120.5, 94	104.5	118.8±11.9 (108–135)	102, 97, 111	112	33	57.5, 55	71.8±7.2 (65–84)
Prerectum length	136.7±20.2 (95–163)	171, 179, 172.5	164	-	105, 99, 97	191	163.7±23 (134–188)	-	137	-	93	127.2±10.8 (110–138)
Rectum length	73.1±4.8 (68–81)	-	67	-	65, 75, 62	-	74.1±6.4 (71–84)	-	75	23	39, 48.5	50.4±6.4 (43–61)
Tail	70.1±7.2 (61–80)	50, 69, 57	71	66	66, 75, 56	67	71.4±3.8 (66–74.5)	66, 65, 66	66	86	76, 87	80.1±11.4 (65–96)
Genital primordium	-	-	-	-	-	-	-	-	-	29.5	53	54.0±14.4 (43–74)
Spicules	-	104, 106, 104	-	106	-	-	-	106, 113, 112	-	-	-	-
Ventromedian supplements	-	25, 25, 27	-	24	-	-	-	24, 28, 26	-	-	-	-

#### Description.

**Figure 14.**
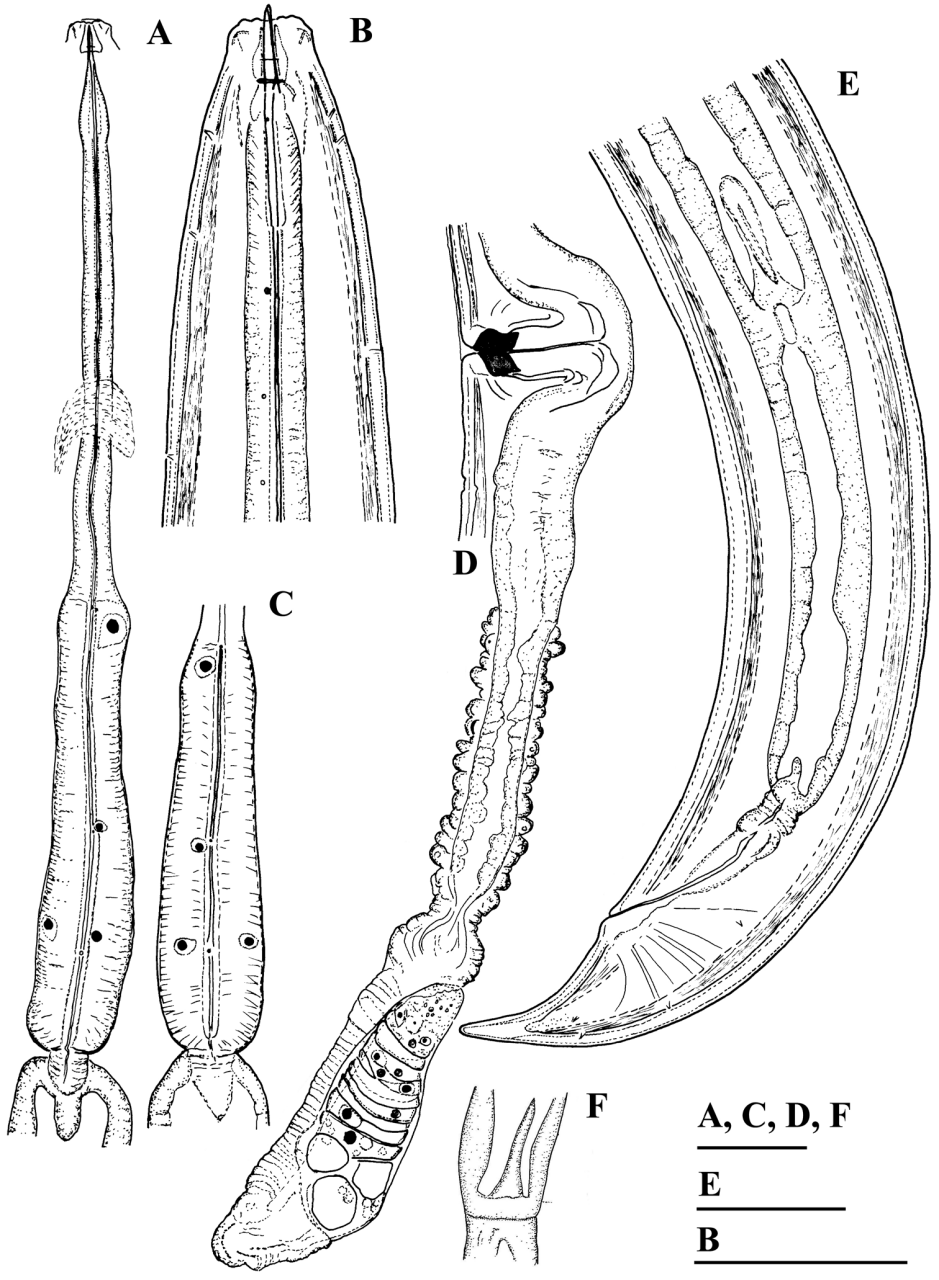
*Pararhyssocolpus paradoxus* (Loof, 1975), gen. n., comb. n. **A, B, D–F** *Female*: **A** Pharyngeal region (KGI) **B** Anterior region (KGI) **D** Posterior genital branch (LI, DM) **E** Posterior end (KGI) **F** Tongue like projection (KGI) *Male* (LI, DM): **C** Pharyngeal expansion. Scale bar: 50 µm (**A–F**).

*Female*. Habitus curved ventrad after fixation, more so in posterior body end. Cuticle smooth, when viewed under light microscope, 3–4 µm thick in postlabial region, 5–7 µm at mid-body and 4–7 µm on tail; consisting of three layers the inner one much ticker and refractive, not reaching the end of tail. Under SEM it is finely transversally striated (annules *ca* 0.6 µm wide). Lip region appears rounded, slightly offset by a depression, 2.3–3.4 times as broad as high, lips amalgamated, outer labial and cephalic papillae protruding above lip region contour. Under SEM inner labial papillae not elevated, close to each other and to oral aperture, outer labial and cephalic papillae below the margin of oral field. Oral aperture seems round hexagonal. Lateral pores well visible (13–14 in the pharyngeal region), the first four as two pairs at the anterior end, next more or less equally spaced. Cheilostom a truncate cone. Amphidial fovea funnel-shaped, opening at level of labial depression, its aperture about half of lip region diam. Odontostyle slender, with clear lumen, aperture subterminal, narrow (Figure 22 B) and indistinct as observed by LM in adults (Figures 18A–C, 19B, 21F); 8–12 times longer than wide, 0.9–1.0 lip region diam. long. Odontophore simple, 1.9–2.3 times odontostyle length long. Guiding ring double, situated at 0.7–0.8 times lip region diam. from anterior end. Nerve ring located at 151–178 µm from anterior end or 32–38% of total neck length. Pharynx consisting of slender but muscular anterior section enlarging gradually and “bibulbar” (Andrássy, 1986), basal expansion with somewhat narrower middle part, 206–231 µm long or 44–52% of total neck length (Figs 14A, 18D). Dorsal nucleus (DN) lying very close to anterior edge of pharyngeal expansion. One nucleus of anterior ventrosublateral pair of pharyngeal glands well visible, large, posterior pair of ventrosublateral nuclei slightly larger, nuclei located almost at one and the same level (pharyngeal characters presented in Table 6). Cardia conoid, measuring 28–39 × 14–19 µm, cell mass near cardia present in some specimens. The posterior end of the intestine with tongue-like projection. Prerectum short, 2–4 times, rectum 1.3–1.8 anal body diam. long. Distinct sphincter at prerectum and rectum junction. Genital system didelphic-amphidelphic, with both branches equally and well developed, anterior 450.5±21.3 (422–478) µm, posterior 463.8±37.9 (404–531) µm long, respectively. Ovaries usually large, oviduct consisting of a tubular part and well developed *pars dilatata*. Sphincter between oviduct and uterus moderately developed. Uterus long (anterior 220–307 µm, posterior 222.5–356 µm long, respectively), bipartite, consists of a wider proximal part followed by narrower distal part surrounded by large hyaline cells. Uteri contain sperm. Vagina extending inwards for 55–74% of body diameter, *pars proximalis* 35–50 × 22–30 µm, with straight walls, *pars refringens* (in lateral view) consisting of two massive trapezoidal separate sclerotised pieces with a combined width of 18–21 µm, *pars distalis* 8.5–12 µm long. Vulva a transverse slit; under SEM vulval lips spindle shaped, irregularities and ruptures of body cuticle present on both sides of vulva. Lateral vulval flaps absent. In two females uterine eggs observed, measuring 133–148 × 68.5–77 µm. Tail conical, ventrally arcuate, distal part offset, tip finger-like, sharply pointed. Three pairs of caudal pores.

**Table 6.** Pharyngeal characters of *Pararhyssocolpus paradoxus* gen. n., n.comb. For abbreviations see Loof and Coomans (1970) and Andrássy (1998b).

Locality	King George Island				Livingston Island			
Characters	KGI1				CDM		DM	
	n	female	n	male	female	male	female	male
DO	2	57, 59	2	54, 56				58
DN=D	8	56–60	3	55, 57, 55	60	57	55	56, 59, 59
Distance DO-DN %	2	1, 0.5	2	1, 1				1
S_1_O	1	78	2	75, 77				78
S_1_N_1_	1	78						
S_1_N_2_	7	75–79	3	75, 76.5, 74			76	76, 77, 79
S_2_O	4	88–91	2	88, 89				89
S_2_N_1_	7	86–89	3	86.5, 87, 87	88	88	87	87, 87, 88
S_2_N_2_	8	87–92	3	87, 87, 88	88	88	87	88, 87.5, 89
AS_1_	1	48						
AS_2_	5	43–49	3	45, 45, 42			46	46, 45, 48
PS_1_	4	69–72	3	70, 70, 70	69	71	70	71, 68, 71
PS_2_	6	70–80	3	71, 70, 73	70	72	70	73, 70, 73

*Males.* General morphology similar to that of female, except for the genital system. Arrangement of pharyngeal gland nuclei presented at Table 6. Genital system diorchic, testes opposed, anterior 318–474 µm (n=3) and posterior 278–436 µm (n=2) long, respectively. Spicules dorylaimid, stout, 1.7–2.6 cloacal body diam. long. Lateral guiding piece with triangular distal part, 19–24 µm long. Sperm oval, measuring 5–9×3–4 µm. Ventromedian supplements contiguous, 24–28 in number, preceded by one adcloacal pair of papillae located at 9–16 µm distance from cloacal opening, out of spicules range; a series of well developed subventral spaced papillae (Jairajpuri and Ahmad 1992) in number 11–18 observed. Post-cloacal papilla present. Tail compared to that in female with narrower finger like tip. Three pairs of caudal pores.

*Juveniles.* Comparison of length of functional and replacement odontostyle and body length yielded in identification of three juvenile stages (second stage juvenile not found). The tail in J1 elongated, sigmoid, in J3 tail elongate with long hyaline extension, ventrally arcuate, sometimes slightly sigmoid, sharply tipped; in J4 ventrally arcuate with gradually tapering distal part, c’ decreases during successive stages to females (Table 5).

**Figure 15.**
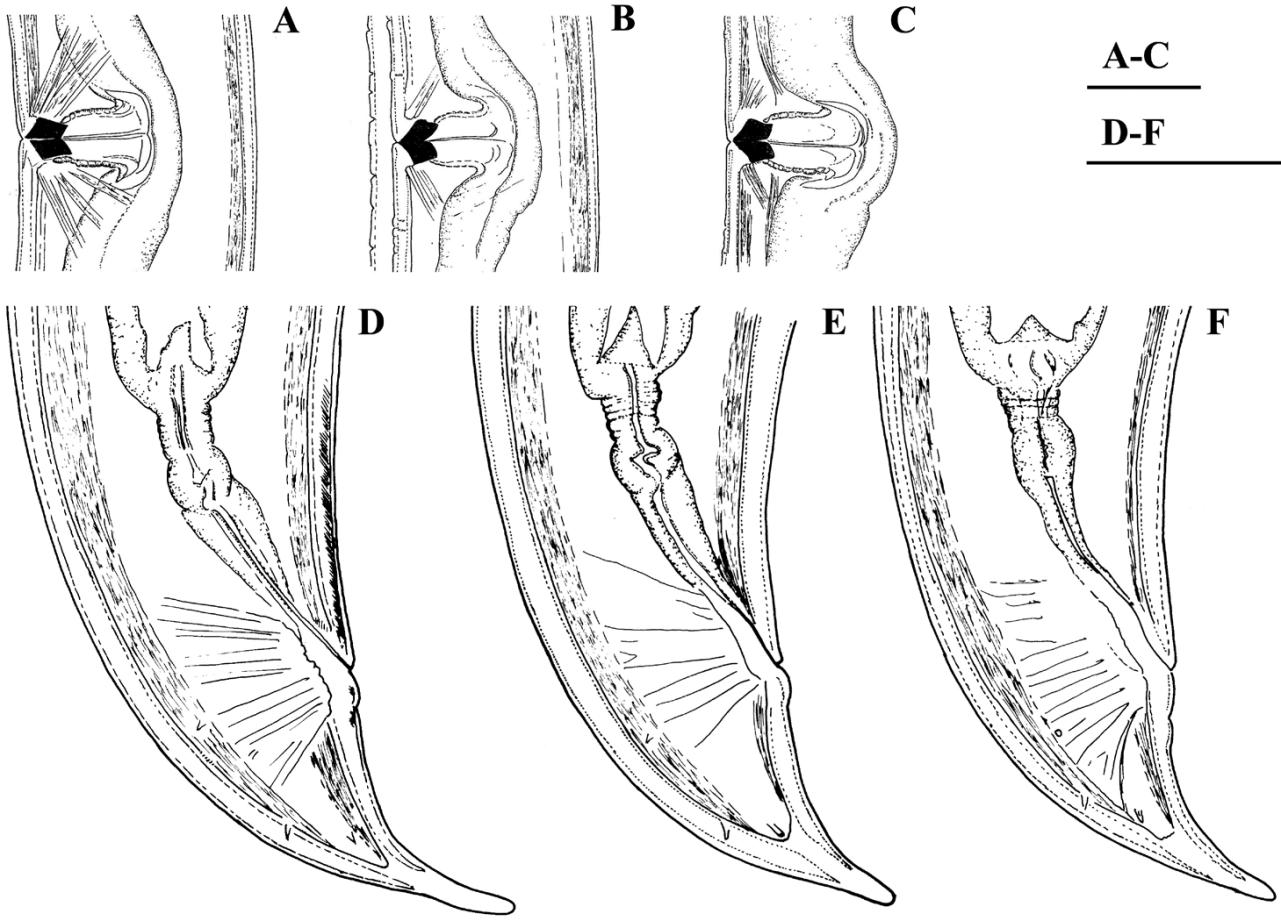
*Pararhyssocolpus paradoxus* (Loof, 1975) gen. n., comb. n. *Female*: **A–C** Vulval region (KGI) **D–F** Tail ends (**D, E** KGI; **F** LI, DM). Scale bar: 50 µm (**A–F**).

**Figure 16.**
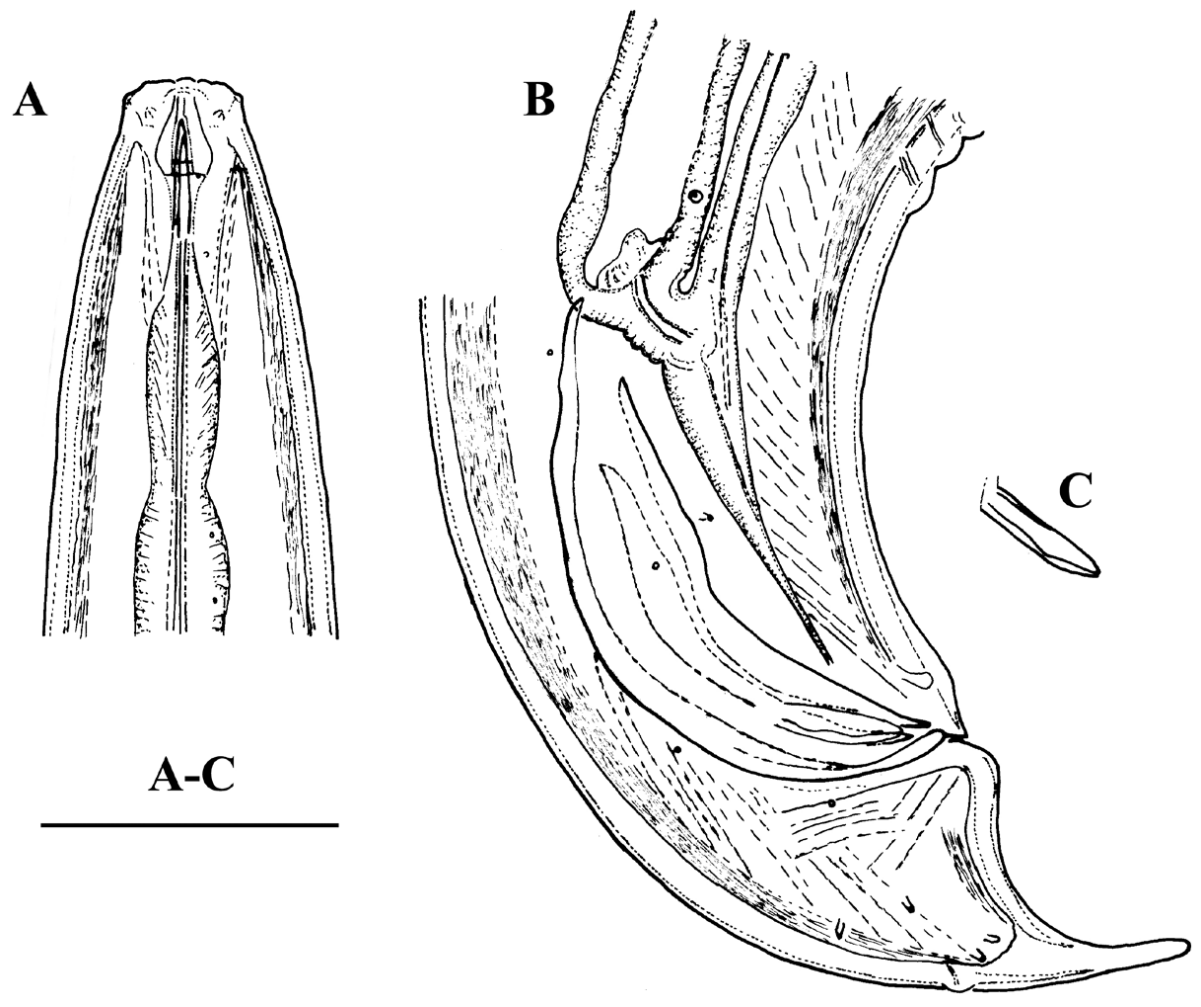
*Pararhyssocolpus paradoxus* (Loof, 1975), gen. n., comb. n. *Male*: **A** Anterior region (LI, DM) **B** Tail end (KGI) **C** Lateral guiding piece (KGI). Scale bar: 50 µm (**A–C**).

**Figure 17.**
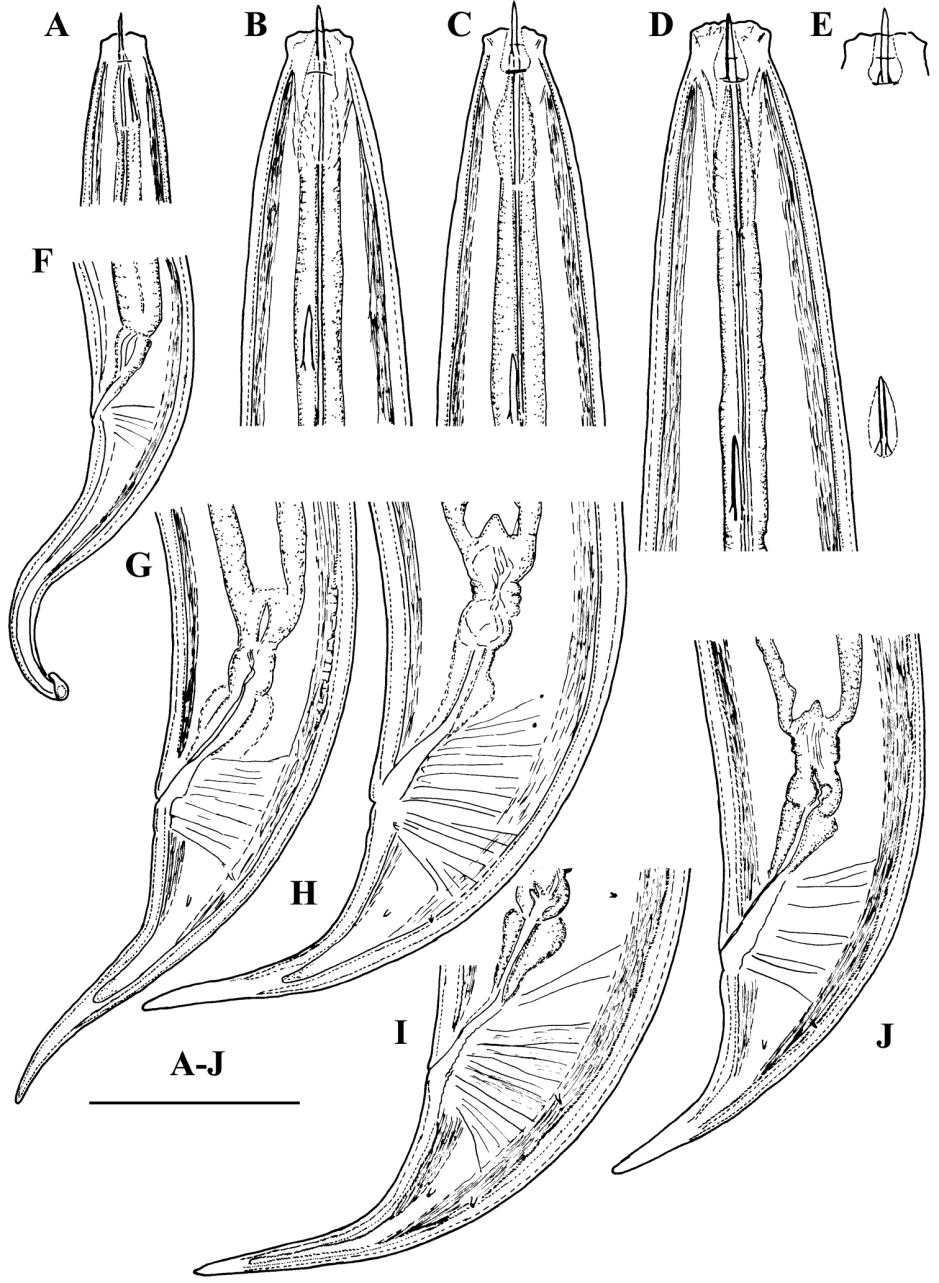
*Pararhyssocolpus paradoxus* (Loof, 1975), gen. n., comb. n. *Juveniles* (KGI): **A–E** Lip region of **A** J1 **B, C** J3 **D, E** J4 **F–J** Tail end of **F** J1 **G, H** J3 **I, J** J4. Scale bar: 50 µm (**A–J**).

**Figure 18.**
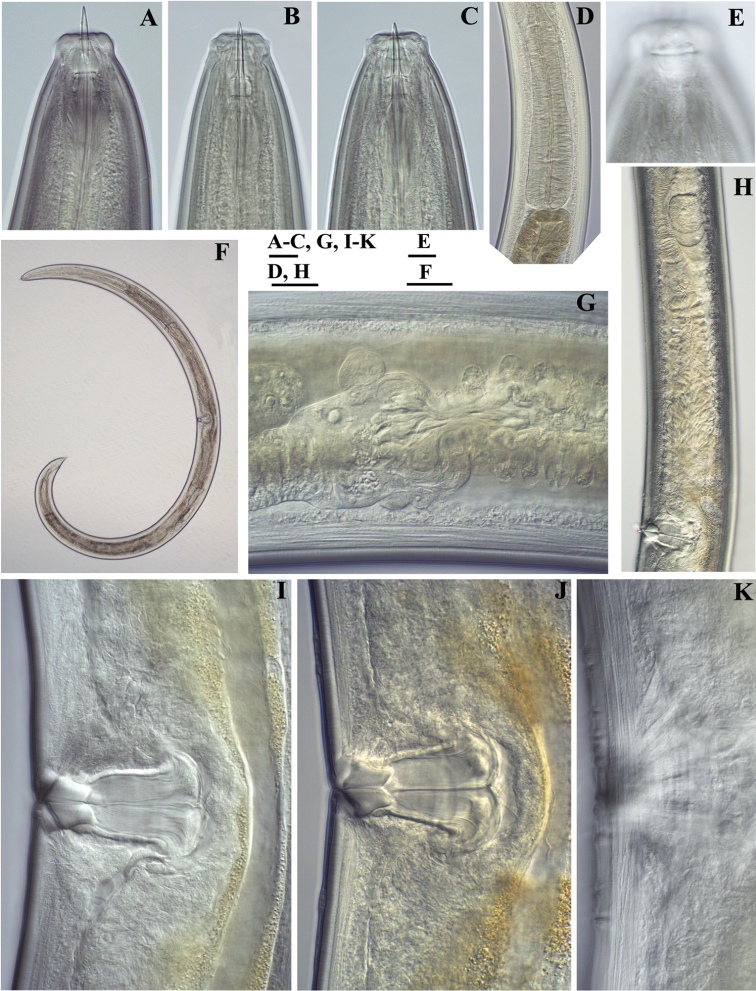
*Pararhyssocolpus paradoxus* (Loof, 1975), gen. n., comb. n. *Female*: **A–C** Anterior region (**A** KGI; **B** LI, DM; **C** NI) **D** Pharyngeal expansion, cardia (KGI) **E** Amphidial fovea (KGI) **F** Entire body (KGI) **G** Sphincter between uterus and *pars dilatata oviductus* (KGI) **H** Anterior genital branch (KGI) **I, J** Vulval region (KGI) **K** Irregularities around vulva (KGI). Scale bars: 10 µm (**A–C, G, I–K**); 50 µm (**D, H**); 6 µm (**E**); 200 µm (**F**).

**Figure 19.**
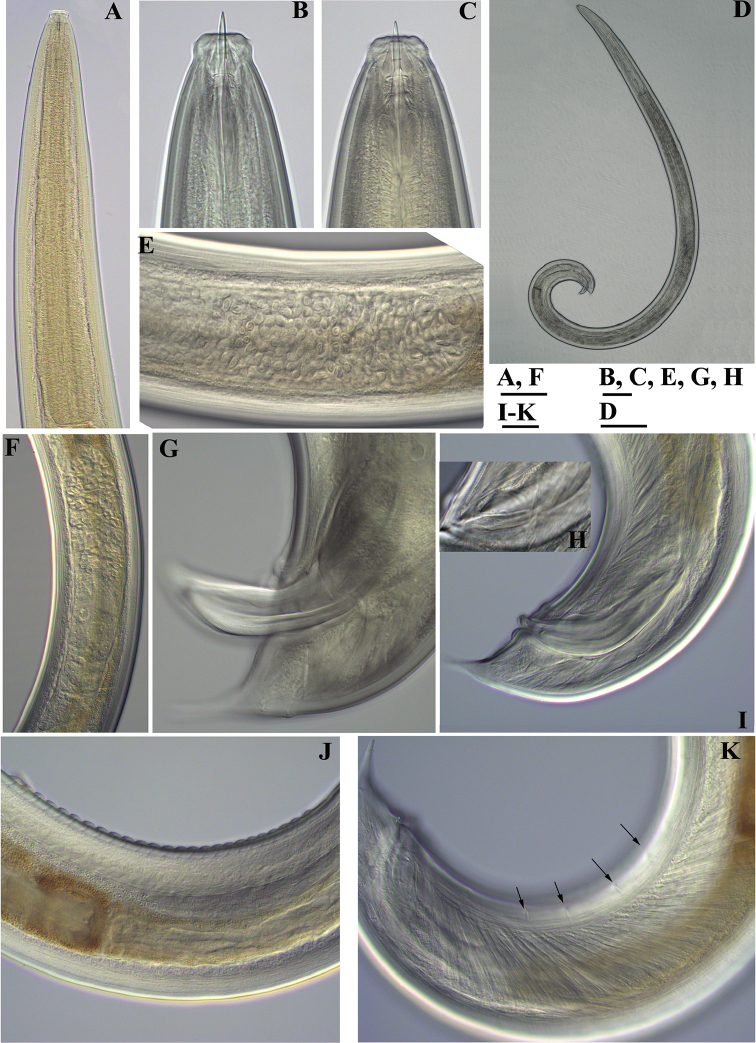
*Pararhyssocolpus paradoxus* (Loof, 1975), gen. n., comb. n. *Male*: **A** Pharyngeal region (LI, CDM) **B, C** Anterior end (**B** KGI; **C** LI, DM) **D** Entire body (KGI) **E** Sperm (KGI) **F** Posterior testis (KGI) **G, I** Spicules (KGI) **H** Lateral guiding piece (KGI) **J** Ventromedian supplements (KGI) **K** Subventral papillae marked by arrows (KGI). Scale bars: 50 µm (**A, F**); 10 µm (**B, C, E, G, H**); 200 µm (**D**); 20 µm (**I–K**).

**Figure 20.**
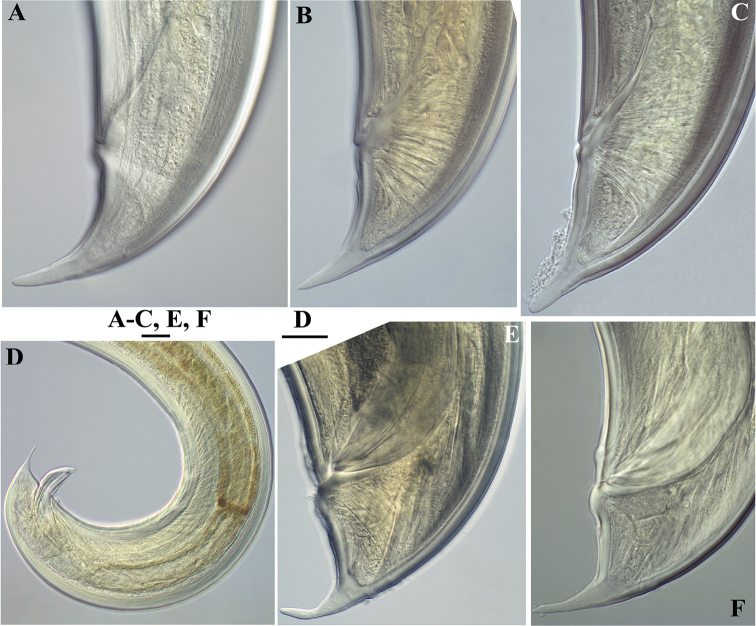
*Pararhyssocolpus paradoxus* (Loof, 1975), comb. n. **A–C** *Female*: **A–C** Tail ends (**A** KGI; **B** LI, DM; **C** NI). **D–F***Male*: **D** Posterior end (KGI) **E, F** Tail ends (**E** CDM, LI; **F** KGI). Scale bars: 10 µm (**A–C, E, F**); 50 µm (**D**).

**Figure 21.**
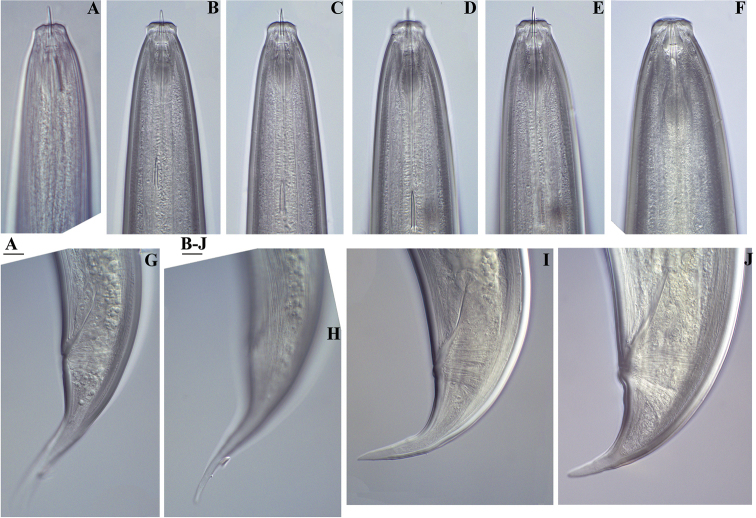
*Pararhyssocolpus paradoxus* (Loof, 1975), comb. n. **A–E, G–I** *Juveniles* (KGI): **A–E** Lip region of **A** J1 **B, C** J3 **D, E** J4 **G–I** Tail end of **G, H** J3 **I** J4. **F, J** *Female*: (KGI): **F** Lip region **J** Tail end. Scale bars: 6 µm (**A**); 10 µm (**B–J**).

**Figure 22.**
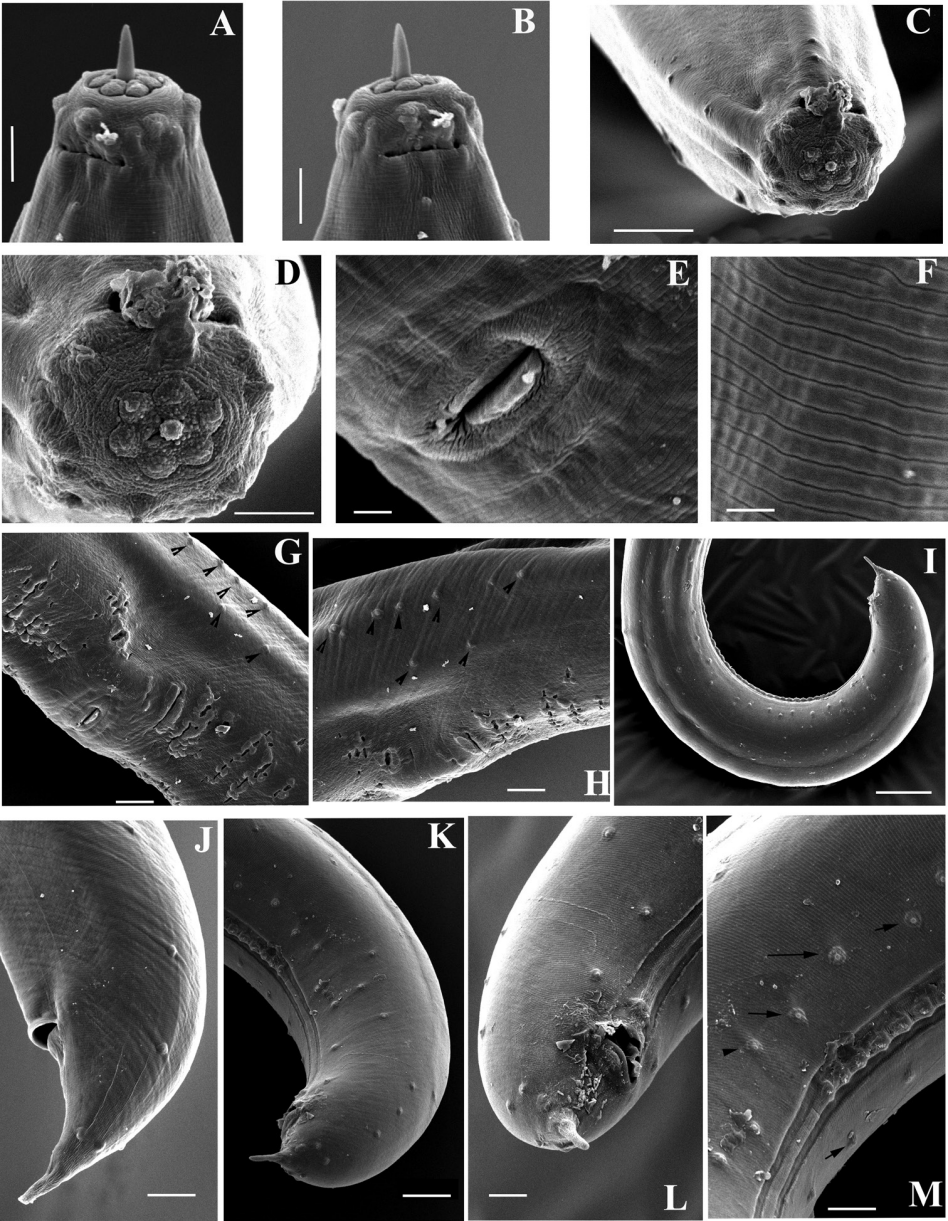
SEM micrographs. *Pararhyssocolpus paradoxus* (Loof, 1975), comb. n. **A–H, J** *Female*: **A–D** Lip region (**A** Sublateral view (NI); **B** Lateral view (NI); **C, D** (LI) In face view) **E** Vulval region (NI) **F** Cuticle striations (NI) **G**, **H** Vulval region, irregularities around vulva, lateral body pores marked by arrows (NI) **J** Tail end (LI) **I, K–M** Male (LI): **I** Posterior end, lateral view **K** Tail end **L** Cloaca **M** Ventromedian supplements and subventral papillae (marked by arrows). Scale bars: 5 µm(**A, B, D**); 10 µm (**C, G, H, J, L, M**); 50 µm (**I**); 20 µm (**K**); 2 µm (**E**); 1 µm (**F**).

**Figure 23.**
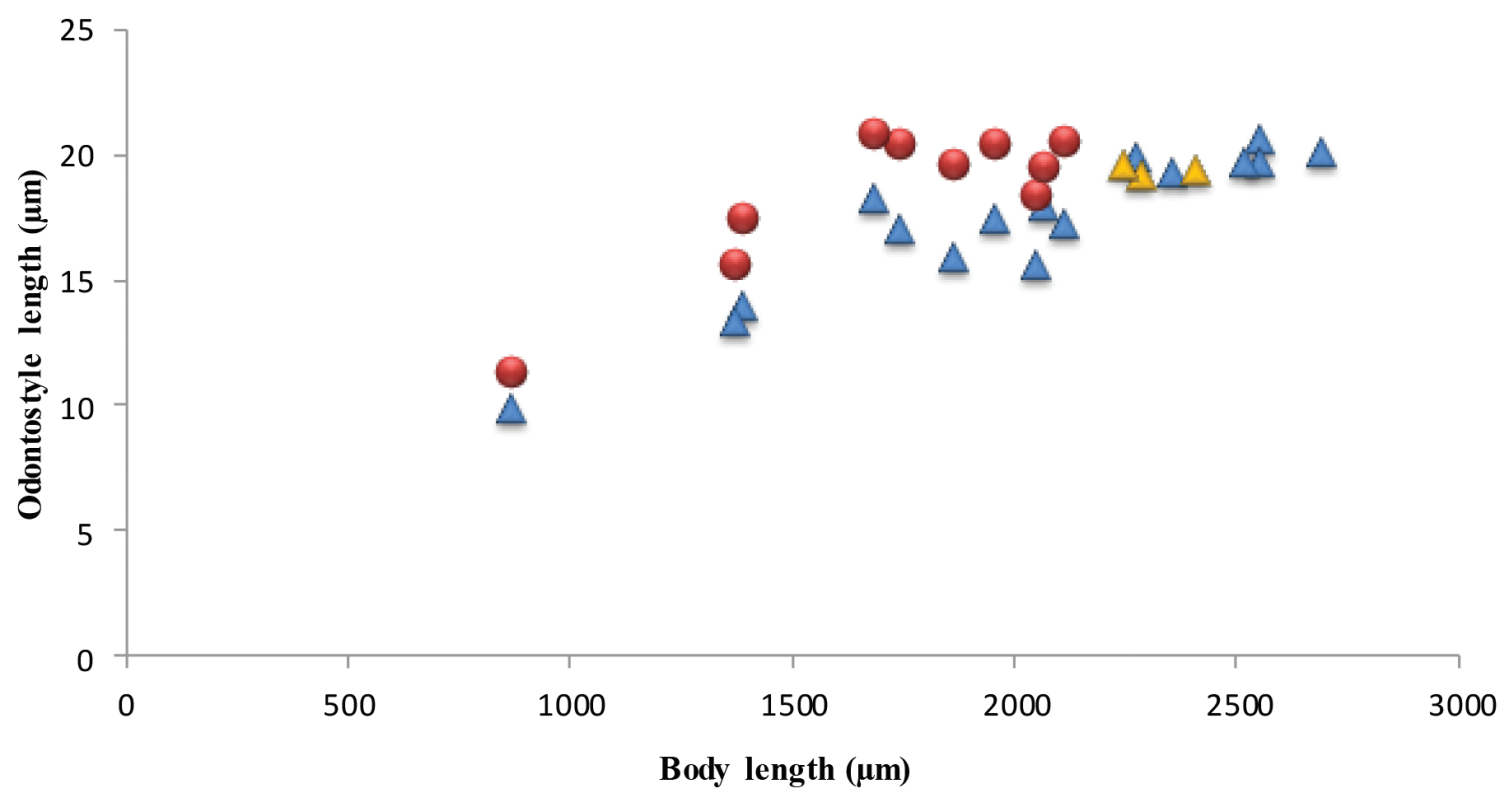
*Pararhyssocolpus paradoxus* (Loof, 1975), comb. n. Scatter plot of the functional (●) and replacement odontostyle (▲) in relation to the body length of the juvenile stages and adults: females (▲) and males (▲).

**Figure 24.**
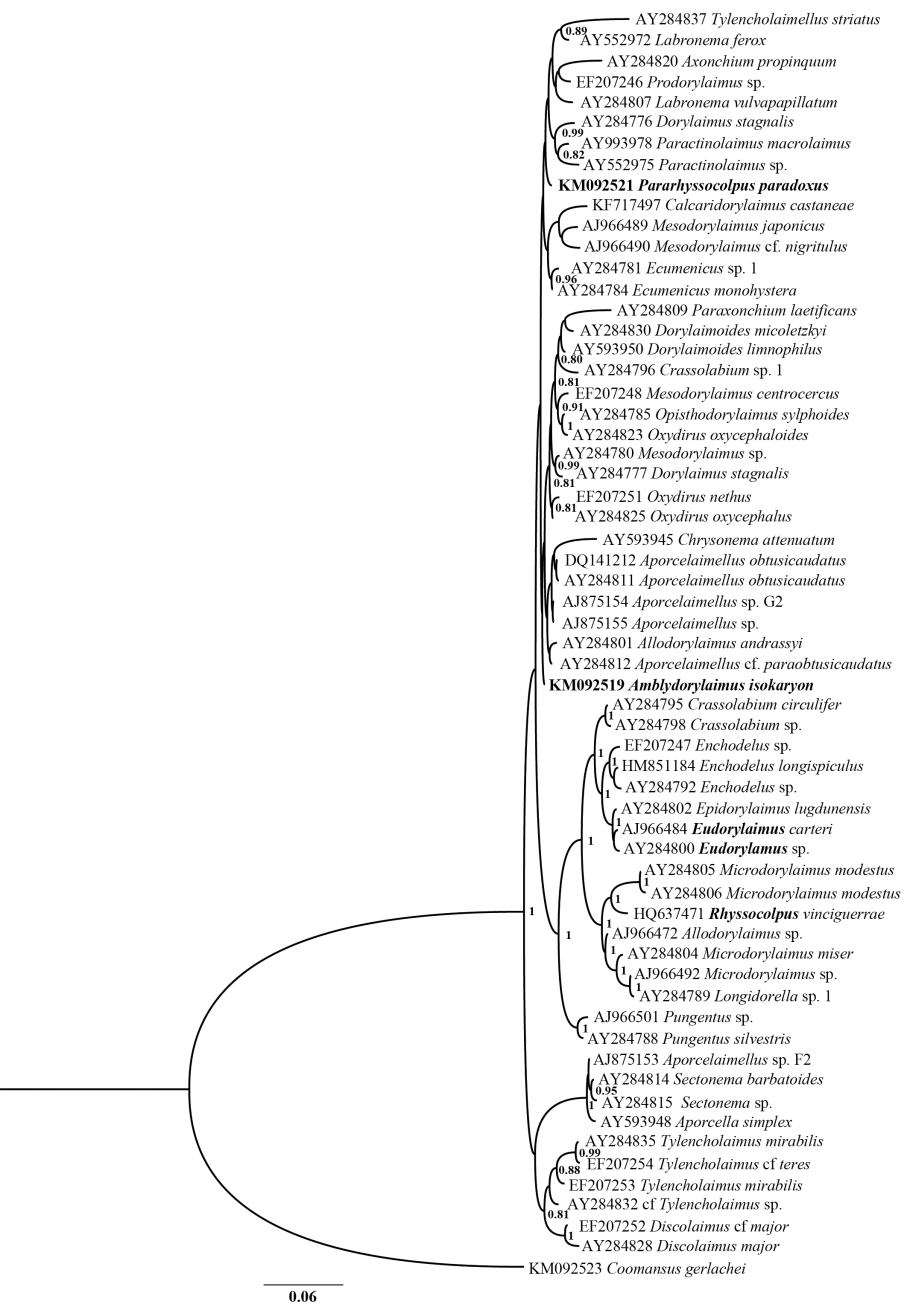
Hypothesis of the phylogenetic relationships of *Amblydorylaimus isokaryon* (Loof, 1975) and *Pararhyssocolpus paradoxus* (Loof, 1975), gen. n. comb. n. based on 18S rDNA (61 sequences) inferred from a Bayesian analysis using GTR+G model and *Coomansus gerlachei* (de Man, 1904) for rooting the tree. Posterior probabilities higher than 0.8 are presented.

**Figure 25.**
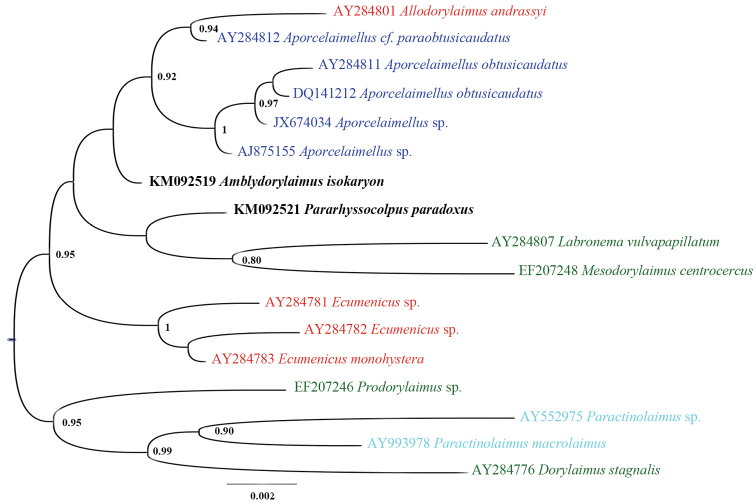
Hypothesis of the phylogenetic relationships of *Amblydorylaimus isokaryon* (Loof, 1975) and *Pararhyssocolpus paradoxus* (Loof, 1975), gen. n. comb. n. based on 18S rDNA of closest species (17 sequences) inferred from a Bayesian analysis using GTR+G model and midpoint rooting of the tree. Posterior probabilities higher than 0.8 are presented. Species coloured according the classification of Andrássy (2009a) and Peña-Santiago and Álvarez-Ortega (2014): dark blue – fam. Aporcelaimidae, light blue – fam. Actinolaimidae, green – fam. Dorylaimidae, red – fam. Qudsianematidae.

**Figure 26.**
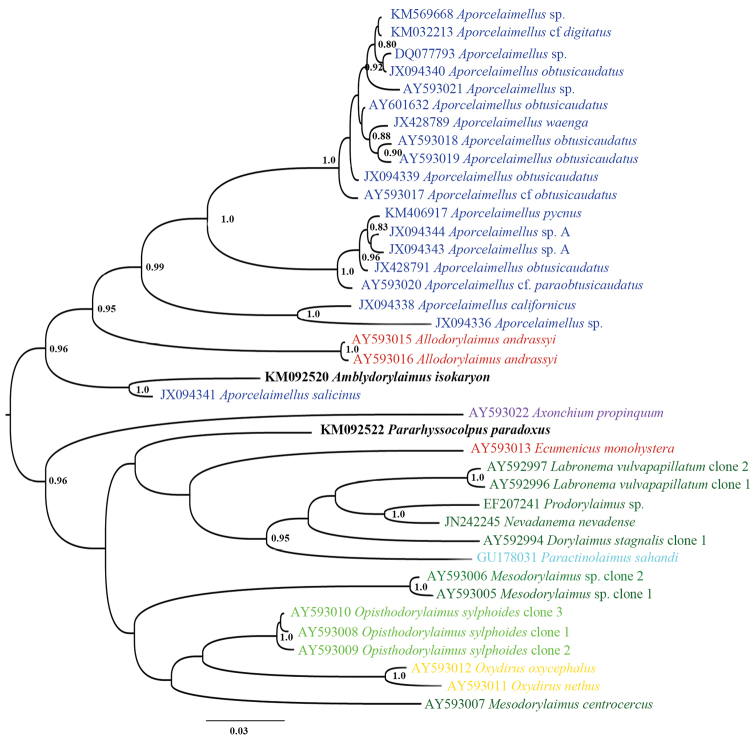
Hypothesis of the phylogenetic relationships of *Amblydorylaimus isokaryon* (Loof, 1975) and *Pararhyssocolpus paradoxus* (Loof, 1975), gen. n. comb. n. based on 28S rDNA D2-D3 inferred from a Bayesian analysis using GTR+G model and midpoint rooting of the tree. Posterior probabilities higher than 0.8 are presented. Species coloured according the classification of Andrássy (2009a) and Peña-Santiago and Álvarez-Ortega (2014): dark blue – fam. Aporcelaimidae, light blue – fam. Actinolaimidae, dark green – fam. Dorylaimidae, light green – fam. Thornenematidae, red – fam. Qudsianematidae, yellow – fam. Swangeriidae, violet – fam. Belondiridae. *For abbreviations of localities see Table 1

#### Sequence and phylogenetic analyses.

The BLAST search using D2-D3 region sequence of *Pararhyssocolpus paradoxus* gen. n., comb. n. showed highest similarity (93%) to the sequences of several *Opisthodorylaimus sylphoides* (Williams, 1959) Carbonell & Coomans, 1985 clones and *Prodorylaimus* sp. (AY593008–10, EF207241, Holterman et al. 2008). The 18S rDNA sequence showed 99% similarity to several dorylaimid species belonging to different families including *Amblydorylaimus isokaryon*, and various *Aporcelaimellus* spp. The hypothesis testing using closely and more distantly related 18S rDNA sequences (Figure 24) revealed distant relationship of *Pararhyssocolpus paradoxus* gen. n., comb. n. to the only available sequences of *Rhyssocolpus* Andrássy, 1971 (*Rhyssocolpus vinciguerrae* Pedram, Pourjam, Robbins, Ye, Peña-Santiago, 2011, Figure 4) (fam. Nordiidae) and *Eudorylaimus* Andrássy, 1959 (two *Eudorylaimus* spp.) (fam. Qudsianematidae). The ambiguous position of both *Pararhyssocolpus paradoxus* gen. n., comb. n. and *Amblydorylaimus isokaryon* could be a result of the low resolution of the SSU rDNA, non-monophyly of these four families and/or probably incorrect species identifications. The majority of the nematode sequences belonging to the superfamily Dorylaimoidea de Man, 1876 available at the GenBank have no morphological and metrical data and their identification is questionable.

In an additional analysis using the most closely related sequences performed in order to clarify the possible evolutionary relationships of *Pararhyssocolpus paradoxus* gen. n., comb. n. (Figure 25): it clustered into the same clade with *Amblydorylaimus isokaryon* and some other species of the families Qudsianematidae, Dorylaimidae and Aporcelaimidae. Further, in the 28S rDNA-based phylogenetic tree *Pararhyssocolpus paradoxus* gen. n., comb. n. grouped with species belonging to different families (Figure 26) and no close relationships to any of them were revealed.

#### Discussion.

The specimens examined generally agree well with data reported for this species, although some differences occurred: lip region offset by slight depression *vs* deep depression; vulva transverse *vs* “probably pore-like rather than transverse”, smaller DN-DO distance (0.5–1 *vs* 1.6–3.4%) (Loof 1975). Further, the distinct sphincter at prerectum/rectum junction, tongue-like structure at the posterior end of intestine and subventral papillae in male were not mentioned in the original description.

Originally this species was attributed to family Qudsianematidae. Loof (1975) placed it in *Eudorylaimus*, because of widened near the middle pharynx and numerous ventromedian supplements. Nevertheless, he reported that it showed several characters close to *Rhyssocolpus* (shape of lip region, short odontostyle, and wrinkled cuticle near vulva, although he regarded the last one a not generic rank character). Subsequently Andrássy (1986) included it in family Nordiidae (genus *Rhyssocolpus*) ignoring the characters in which this species differs from the other members of genus *Rhyssocolpus* e.g. the greater number of contiguous ventromedian supplements and specific shape of pharyngeal expansion. Again, Loof (1988) reported that many features of this species (numerous and contiguous supplements, pharyngeal expansion at about half pharynx length, DN lying at about 60% of pharynx, distinct first pair of ventrosublateral pharyngeal glands) conflicted with the diagnosis of *Rhyssocolpus* and continued to regard this Antarctic species as a member of *Eudorylaimus* (Qudsianematidae). Very recently, Peña-Santiago et al. (2015) provided a revised taxonomy of the genus *Rhyssocolpus* and proposed *Rhyssocolpus paradoxus* be retained under *Eudorylaimus*. However, it differs from the latter genus by the arrangement of ventromedian supplements in males (contiguous *vs* spaced), double *vs* single guiding ring, slender *vs* wider odontostyle and specific shape of pharyngeal expansion.

Recent molecular studies (Holterman et al. 2008; Pedram et al. 2011; Peña-Santiago et al. 2015) as well as our molecular data inferred from the analysis of 18S and D2-D3 expansion segments of the 28S rDNA, showed that this genus could not be assigned to any known Dorylaimoidea family.

With considering the differences discussed above, as well as molecular data, the herein studied species cannot be regarded either as a member of the genus *Rhyssocolpus* or the genus *Eudorylaimus* and their attributed families, consequently a new genus *Pararhyssocolpus* gen. n., and a new family Pararhyssocolpidae fam. n. are proposed to accommodate this species.

### Family Pararhyssocolpidae fam. n.

Taxon classificationAnimaliaDorylaimida

http://zoobank.org/B2D2F40F-283F-41A6-A2C4-5DFD3461EB80

#### Diagnosis.

Dorylaimoidea. Nematodes of a medium sized body, 2–3 mm. Cuticle dorylaimoid, finely transversally striated. Lip region rounded, inner labial papillae distinct, not elevated, amalgamated and close to oral aperture, outer labial and cephalic papillae below the margin of oral field. Odontostyle slender, shorter than or as long as labial diameter, with narrow aperture, indistinct under LM and clear lumen. Odontophore simple. Guiding ring double. Pharynx occupying about half total pharynx length. Female genital system didelphic-amphidelphic, uterus bipartite, *pars refringens vaginae* well developed, vulva transverse. Irregularities and ruptures of body cuticle around vulva present. Spicules dorylaimoid, lateral guiding piece simple. Ventromedian supplements contiguous, numerous. Tail similar in both sexes, conical, ventrally arcuate, distal part long, finger-like. First stage juvenile with long sigmoid tail.

### Pararhyssocolpus gen. n.

Taxon classificationAnimaliaDorylaimidaPararhyssocolpidae

http://zoobank.org/95A47B1D-D7A7-4379-ABC9-EDF1A227AED1

#### Diagnosis.

With characters of the family.

#### Relationships.

On the basis of main characters, this genus/family appears close to family Nordiidae, Qudsianematidae (subfamily Qudsianematinae Jairajpuri, 1965) and Dorylaimidae. The new family differs from the first family in pharynx widening at the middle of neck *vs* pharynx widening behind the middle of the neck, the pharyngeal expansion shape (somewhat “bibulbar”, with narrower middle part *vs* cylindrical), ventromedian supplements contiguous *vs* mostly spaced (except *Lenonchium* Siddiqi, 1965, it differs from the new family by its longer and filiform tail). From subfamily Qudsianematinae, Pararhyssocolpidae fam. n. can be differentiated by its double *vs* single guiding ring and labial papillae arrangement (small *vs* larger distance between inner labial papillae), indistinct *vs* distinct aperture of odontostyle. Also, the new family differs from fam. Dorylaimidae in odontostyle aperture (indistinct *vs* distinct) and especially in its characteristic postembryonic development pattern – J1 with long tail, c’ decreasing in successive stages and adults caused by the increasing of anal diameter rather than shortening of tail, adults with similar tail shape - conical with distal third much narrower, finger-like *vs* one or more juvenile stages bearing long (filiform or conical elongated) caudal region, adults with similar (either long or short and rounded, never conical) or dissimilar (long in females, short and rounded, exceptionally conical, in males) tail (Peña-Santiago and Álvarez-Ortega 2014). Recent studies based on molecular data (Holterman et al. 2008; Pedram et al. 2011; Álvarez-Ortega et al. 2013a, c; Peña-Santiago et al. 2015) show that the current classification of superfamily Dorylaimoidea is questionable with most families being not monophyletic taxa, as some of the genera are closer to members of other families. Further integrative studies are needed to clarify its phylogeny and systematics and to understand which characters are homologous and which are the results of convergent or parallel evolution (Vinciguerra et al. 2014).

#### Distribution.

This species (genus, family) is endemic in Maritime Antarctic, having been recorded from many islands: Signy (Loof 1975; Maslen 1979; 1981), Coronation, Elephant, Intercurrence, Galindez, Blaiklock, Limpet (Loof 1975; Maslen 1979), Guébriant (Maslen 1979), Adelaide (Maslen and Convey 2006; Newsham et al. 2006), Anchorage, Leonie (Maslen and Convey 2006), Livingston (Peneva et al. 2009), Francis (Velasco-Castrillón and Stevens 2014) and King George Islands (Kito 2009; Russell et al. 2014). This is the first report of the species from Nelson Island.

## Supplementary Material

XML Treatment for Amblydorylaimus isokaryon

XML Treatment for Pararhyssocolpus paradoxus

XML Treatment for Pararhyssocolpidae

XML Treatment for Pararhyssocolpus
